# Prioritising Indicators for Large-scale Monitoring and Assessment of Food Environments for Public Health

**DOI:** 10.1007/s13679-026-00705-8

**Published:** 2026-04-22

**Authors:** Lana Vanderlee, Kelly Garton, Mavra Ahmed, Kathryn Backholer, Carolina Batis, Marie A. Bragg, Emma Boyland, Adrian J. Cameron, Luciana Castronuovo, Elizabeth K. Dunford, Clara Gómez-Donoso, Guanlan Hu, Alexandra Jones, Bridget Kelly, Amos Laar, Meron Lewis, Christiam Méndez-Lazarte, Sally Mackay, Christina M. Pollard, Erica Reeve, Sally Schultz, Tailane Scapin, Suzanne Soares-Wynter, Rina Swart, Boyd Swinburn, Caroline Vaillancourt, Gary Sacks

**Affiliations:** 1 School of Nutrition and Centre NUTRISS, Université Laval, Québec, QC Canada; 2 Faculty of Medical and Health Sciences, School of Population Health, University of Auckland, Auckland, New Zealand; 3 Department of Nutritional Sciences and Joannah and Brian Lawson, Centre for Child Nutrition, Temerty Faculty of Medicine, University of Toronto, Toronto, ON Canada; 4 Institute for Health Transformation, Global Centre for Preventive Health and Nutrition, Deakin University, Geelong, Australia; 5 Center for Nutrition and Health Research, National Institute of Public Health, Cuernavaca, Morelos Mexico; 6 Department of Population Health, New York University Grossman School of Medicine, New York, NY USA; 7 Department of Psychology, Institute of Population Health, University of Liverpool, Liverpool, UK; 8 Fundación Interamericana del Corazón (FIC), Buenos Aires, Argentina; 9 Gillings Global School of Public Health, Department of Nutrition, The University of North Carolina, Chapel Hill, NC USA; 10 The George Institute for Global Health, Food Policy Division, University of New South Wales, Sydney, NSW Australia; 11 Open University of Catalonia, Barcelona, Spain; 12 Data Sciences Institute, Columbia University, New York, NY USA; 13 Faculty of Science, Medicine and Health, School of Medical, Indigenous and Health Sciences Wollongong, University of Wollongong, Wollongong, Australia; 14 School of Public Health, Department of Population, Family and Reproductive Health, University of Ghana, Legon, Accra, Ghana; 15 Faculty of Medicine, School of Human Movement and Nutrition Science, University of Queensland, Brisbane, QLD Australia; 16 Universidad de Lima, Instituto de Investigación Científica PE, Lima, Peru; 17 Faculty of Health Sciences, Curtin School of Population Health, Curtin University, enABLE Institute for Health Science and Curtin Medical Research Institute Curtin University, Perth, WA Australia; 18 Caribbean Institute for Health Research, Tropical Metabolism Research Unit, University of the West Indies, Kingston, Jamaica; 19 Department of Dietetics and Nutrition, University of the Western Cape, Cape Town, Western Cape South Africa

**Keywords:** Food environments, Food policy, Nutrition policy, Food environment monitoring, Indicator framework

## Abstract

**Purpose of Review:**

This study aimed to prioritise key indicators for monitoring and assessing the healthiness of food environments, including at the country level. We conducted a literature review and engaged a consortium of researchers from the International Network for Food environments Research, Monitoring and Action Support (INFORMAS) as part of a prioritisation process.

**Recent Findings:**

We identified 375 existing indicators that had been used to measure food environments. Researchers were consulted on priorities and implementation considerations through a series of interviews, focus groups and an online survey. Sixteen indicators were short-listed for prioritisation, and five indicators were identified as key priorities: proportion of packaged food and drinks products classified as unhealthy; frequency of exposure to unhealthy food promotion; price of a healthy diet and the price of the current diet as a proportion of income; proportion of schools in which unhealthy foods are provided or available for sale regularly; and application of back of package and front of package food labelling.

**Summary:**

This study lays the groundwork for standardised and scalable food environment monitoring that can drive policy action to support healthier food environments globally. Future areas for inquiry include tools to classify healthy foods and outlets, which could be adapted across policy areas, and the establishment of evidence-informed benchmarks for assessment of each indicator.

**Supplementary Information:**

The online version contains supplementary material available at 10.1007/s13679-026-00705-8.

## Introduction

Unhealthy diets are a leading contributor to obesity and poor health globally [1]. The unhealthy state of population diets is driven by food environments that are dominated by unhealthy foods that are highly accessible and heavily promoted [2]. Swinburn et al. define food environments as the physical, economic, policy and sociocultural surroundings, opportunities and conditions that influence dietary patterns [3]. Creating healthier food environments is central to population-level progress on reducing noncommunicable diseases (NCDs). Policy actions that address key characteristics of food environments have been identified by various United Nations agencies including the World Health Organization (WHO) and others as cost-effective ways of supporting healthier dietary patterns [4, 5]. Monitoring of food environment characteristics is critical to understand the current state of food environments and trends over time. Data from monitoring can inform government policy and interventions aimed at creating healthier food environments, including evaluation of their impact.

In 2013, the International Network for Food environments Research, Monitoring and Action Support (INFORMAS), published a series of protocols to measure and monitor key elements of food environments across seven strategic domains (food composition, food labelling, food promotion, food provision in public sector settings, food prices, food retail and food trade and investment). As of 2025, INFORMAS methods [6, 7] have been implemented across more than 65 countries and all WHO regions, and have been used to facilitate comparisons of food environments between and within countries [6–8]. Despite efforts to standardise monitoring of food environments as part of INFORMAS, the wide range of methods, tools and indicators available, coupled with varying resources available for monitoring in different contexts, has meant that food environment monitoring efforts have been inconsistent, with large variations in the type and volume of data collected and analysed. Furthermore, dynamic, evolving food environments and innovative research questions have resulted in alternate definitions, conceptual frameworks and methods to measure food environments [9, 10], contributing to further variations in measurement approaches and data. Greater standardization of methods and the prioritisation of indicators is likely to ensure greater focus on the most critical elements of food environments and increase the ability to systematically compare data across different countries and over time [11]. The establishment of a key set of indicators for monitoring the healthiness of food environments across countries is particularly important given the global nature of current food systems that are dominated by multi-national food companies [12, 13]. Identifying specific indicators of particular importance could also enhance the feasibility of conducting regular, systematic monitoring and surveillance activities, particularly in low-resource settings.

There is currently limited systematic surveillance of food environments (defined as the routine, systemic collection, analysis and interpretation of food environments data to plan, implement and evaluate public health performance [14]) by governments. To our knowledge, no country has established a comprehensive government-led monitoring system for food environments. This may be, in part, due to competing political priorities, lack of standardised guidance, perceived challenges with feasibility, the commercial nature of some of the available data, and perceived costs of monitoring at large scale (e.g., national level) and on an ongoing basis. Similarly, there have been limited efforts to collate and synthesize available data at national and international levels.

The Food Systems Dashboard [15, 16] (FSD) is one example of global surveillance and monitoring of food systems. The FSD incorporates a relatively small number of indicators related to food environments, focused on food availability data and food purchasing data that are routinely collected by global agencies, such as the food balance sheet data from the Food and Agriculture Organization of the United Nations (FAO), or from industry data at an aggregated level for each country (e.g., using the Euromonitor Passport database). While the FSD incorporates data from a large number of countries, the food environment data presented in the FSD do not currently assess all of the critical elements of food environments, such as the nutritional quality of the food supply, affordability of a healthy diet, marketing or labelling [16, 17]. Country-level food environment dashboards have been developed by researchers, in collaboration with policy makers, in Australia and New Zealand [18, 19]. These dashboards have highlighted potential ways to synthesise and communicate data on the relative healthiness of food environments at the national level. Indicators included in those dashboards were largely based on what data were already available in those countries via research-related projects, with only a small amount of data available through routine government-led surveillance.

To provide methodological support for research to address the identified gaps in food environment monitoring, this study aimed to prioritise key indicators for monitoring and assessment of the healthiness of food environments, including at the country level.

## Methods

We used the INFORMAS conceptual framework to structure our review, focusing on the seven domains related to characterising the consumer food environment: food composition, food promotion, food labelling, food retail, food prices, food provision, and food trade and investment [3]. Other INFORMAS domains (upstream public-sector [20], private-sector policies and actions [21], and downstream population diet quality [22]) were not included as they do not directly shape individuals’ exposure to or interaction with food environments and/or require different types of data collection (namely, policy analyses and individual-level data).

We used a multi-stage expert consensus process to iteratively develop a proposed set of prioritised food environment indicators. The prioritisation process was guided by members of the INFORMAS research community, including co-authors of this paper [6]. INFORMAS has established leadership teams and coordinators for each domain that consist of international experts in the field representing diverse geographic and cultural regions of the world. Leadership teams were involved at multiple stages of the process (described below) to support identification of potential indicators within their domain, and the development of a short-list of important indicators for further feedback and prioritisation. The broader INFORMAS membership, consisting of researchers and practitioners involved in food environment research and policy globally, were also invited to provide feedback on the prioritisation of indicators and implementation considerations through an online survey.

The prioritisation process consisted of three stages: (1) a narrative review of existing indicators, validated with input from domain coordinators; (2) focus groups and interviews with researchers from the domain leadership teams to supplement the review, short-list indicators for prioritisation, and understand contextual factors related to monitoring; and (3) a broader survey with a larger group of researchers and practitioners to get input on priorities and implementation considerations.

**Fig. 1:**
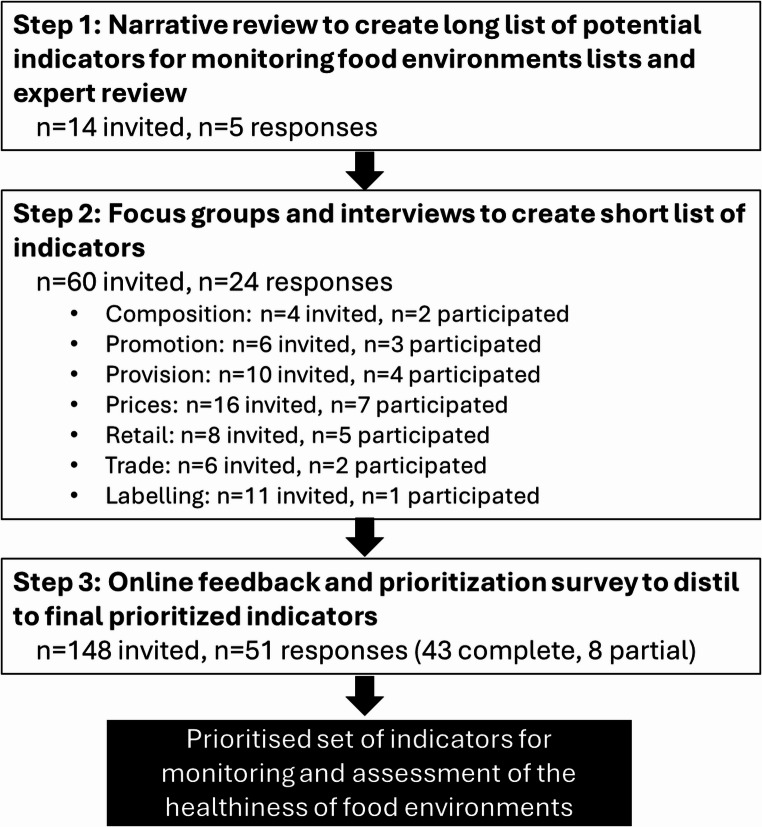
Flow chart of steps in the research process, including the number of participants that were invited and participated in each step of the process

A flow chart documenting participation in all steps of the study is shown in Fig. 1.

This study was reviewed and received ethics clearance from the research ethics board at Université Laval (File Number 2022 − 496/11-01−2023) and Deakin University (Project Number HEAG-H 10_2023).

### Step 1. Narrative Review of Food Environment Indicators to Develop a Long-list of Indicators

To identify potential indicators for inclusion, we conducted a broad narrative review of food environment indicators. Our starting point was the foundation INFORMAS methods papers published in 2013 [2, 3, 22–30], protocols listed on the INFORMAS website [31–35], food environment dashboards [16, 18, 19], and other prominent international food environment monitoring initiatives known to the authors [9, 36–43] as well as several unpublished protocols or reports, for a total of 34 sources. We then examined papers that cited these sources. Within identified papers, we identified a total of 375 indicators and potential outcomes of interest that were reported. Only articles in English and French were included. A long-list of these indicators was shared with domain leadership team coordinators (*n* = 14 invited, *n* = 5 responded), who were asked to identify additional indicators from their own work or work with which they were familiar as well as articles or reports published in languages other than English and French.

### Step 2. Focus Groups and Interviews

A total of 60 individuals from research and non-governmental organizations comprising the seven INFORMAS domain leadership teams were invited to participate, and 25 provided consent and participated. Participants were asked to review the long-list of indicators and then participated in focus groups or interviews designed to propose a small number of these indicators (or indicators that combined previously identified indicators) as highest priority within their domain (short-list). We developed proposed criteria to guide the selection of priority indicators (refer to Box 1) based on our experience working in this field. Proposed criteria were developed related to the indicators’ relevance for public health, feasibility of data collection, sensitivity to change, and ease of interpretation. Participants were asked if they had any feedback on these criteria and were prompted to add any additional criteria.

**Box 1**. Proposed criteria for identifying priority indicators for use in monitoring and assessment of the healthiness of food environments at the country level

**Table:** 

1. How likely is it that the indicator will influence dietary patterns?
2. How feasibly can data for the indicator be collected and analyzed at scale?
3. How sensitive will the indicator be to changes over time?
4. How likely is the indicator to change in response to policy change?
5. How easily can the indicator be communicated to the general public and government stakeholders?
6. Is the indicator likely to allow for international comparisons?

### Step 3. Online Survey with INFORMAS Network

An online survey was conducted in May and June 2023. Food environment researchers and practitioners who had participated in recent INFORMAS activities, beyond the INFORMAS leadership members, were contacted and invited to participate in the survey. A total of 148 individuals from 45 countries were sent email invitations, and 51 provided written consent and completed at least some of the survey.

For each domain, the survey presented participants with a short-list of indicators (from Step 2). Participants were asked to provide open-ended comments or recommendations for each indicator and related implementation considerations, and to make suggestions for additional indicators. Next, participants were asked to prioritise the top 5 to 7 indicators across all domains, using the explicit criteria outlined in Box 1. Participants could select multiple indicators within a single domain.

Survey participants were also asked about two cross-cutting considerations that were relevant for most or all domains: criteria to classify the relative healthiness of foods, and application of benchmarks (i.e., ratings for ‘good’ or ‘poor’ performance). In relation to systems for classifying the healthiness of foods (which is a central component of assessing many facets of food environments), participants were presented with a proposed approach that incorporated three classification systems (a country-specific system, the Nova system and the Health Star Rating) that could be used for monitoring, potentially in combination. We proposed this multi-system approach in acknowledgement of the lack of consensus regarding the most appropriate nutrient profiling system across different contexts [44–46], the prevalence of a large number of national and regional systems that support policy implementation [45], and the desire to provide at least some degree of international comparisons using a common classification system (in this case, the Health Star Rating [47]). Participants were asked to comment on the appropriateness of the proposed food classification approach. In relation to benchmarks for indicating performance, we provided examples of how benchmarks could be used to add an interpretive element to the indicators selected and requested feedback on the proposed approaches.

The focus groups, interviews and survey results were supplemented by feedback on the short-list of indicators received at an informal workshop run by INFORMAS at the World Public Health Nutrition Congress in June 2024 that convened global public health experts.

### Analysis

Open-ended comments (verbal and written) received from co-authors, invited experts and researchers throughout the research process and in the congress workshop were systematically considered by LV and GS in relation to each draft indicator. Feedback was aggregated (e.g. common concerns, suggestions) and used to adjust the indicators and improve clarity in an iterative process. Implementation considerations for each domain and overall were summarised.

The results of the online survey prioritisation exercise were summarised by assigning each indicator a score based on its ranking position (e.g., rank 1 received the highest score and rank 7 the lowest), and the frequency of being ranked in the top seven. To visualize these results, we produced horizontal stacked bar charts showing the distribution of rankings assigned to each indicator. The short-list of proposed indicators was continuously refined in response to open-ended feedback provided in focus groups, interviews and survey, and with multiple rounds of consultation between LV, GS and the broader authorship team.

The final set of prioritised indicators was selected based on the results from the ranking task, the open-ended feedback received from participants and the authorship group, and consideration of the underlying data sources to inform the indicator. We also endeavoured to ensure the inclusion of indicators across multiple domains.

## Results

The long-list of the 375 potential indicators identified from Step 1 can be found in Online Resource 1.

### Participant Characteristics

Participant characteristics from Steps 1 and 2 are not described to protect confidentiality, given the small sample size. In the online survey in Step 3, participants’ affiliate institutions were based in all WHO regions (Western Pacific *n* = 20; Americas *n* = 14; Europe *n* = 8; Africa *n* = 3; Eastern Mediterranean *n* = 2; South-East Asia *n* = 2, Unknown *n* = 2). When asked what INFORMAS domains they had been involved with or methods they had used in the past, participants were most frequently familiar with the food promotion (40%), food retail (34%) and food labelling (32%) domains, followed by food composition (24%), food prices (16%), and food provision (14%), with only one having experience with the trade and investment domain (2%). In addition, 32% of participants indicated that they had been involved with or used the public sector domain and 24% the private sector domain.

### Short-List of Indicators

Table 1 describes the refined short-list of 16 indicators that were identified in Step 2, incorporating feedback on content and framing from open-ended feedback in Step 3 and from the workshop and authorship team.

**Table 1:** Short-list of indicators for monitoring and assessment of food environments

DOMAIN	Indicator
Composition (COMP)	COMP1* Proportion of packaged food and drinks products that are classified as: **1a**. Unhealthy according to a national nutrient profiling system *and/or* **1b**. Ultra-processed according to Nova
	**COMP2** Proportion of packaged food and drinks that meets relevant government (re)formulation targets for nutrients to limit (e.g. sodium, fat, total or added sugars, trans fat)
	**COMP3** Mean level of saturated fat, trans fat, total or added sugars and sodium in packaged food and drinks (per 100 g or 100 ml)
	COMP4* Proportion of food and drinks available from major fast-food chains classified as: **4a.** Unhealthy according to national nutrient profiling system and/or **4b**. Ultra-processed according to Nova
Price (PRICE)	**PRICE1** Food prices: Changes in the price of key healthy food and drinks relative to changes in the price of key unhealthy food and drinks
	**PRICE2** Diet costs: Differential between the cost of healthy diets (recommended in national dietary guidelines) relative to the cost of current diets
	**PRICE3** Diet affordability: Cost of a healthy diet relative to the cost of a current diet, as a proportion of income of a reference household or relative to total income (by income level and/or other key measures of socio-economic status)
Promotion (PROMO)	**PROMO1** Recommend identifying at least *2 priority media/settings* to monitor from the list below, with indicators identified for each media/setting: **1a. Television advertising**: Average number of unhealthy food ads (product and brand-based) per hour across the day (e.g., 6am to 10pm) on channels that have or are likely to have substantial child audiences **1b. Digital advertising**: Number of unhealthy food ads (product and brand-based) per hour of screen-time **1c. Outdoor advertising**: Number of unhealthy food ads (product and brand-based) in key public transport settings (e.g., bus/train stations, bus/stops, bus shelters likely to be frequented by children, such as on major routes to schools/city centres) (by geographic location) **1d. Elite sport sponsorship**: Number of elite sports teams that have an unhealthy food brand as a major sponsor **1e. Community sport-based advertising**: Number of unhealthy food ads (product and brand-based) at community sport, recreation and leisure settings (by geographic location) **1f. Product packaging**: Proportion of unhealthy food and drinks that have promotional elements that appeal to children **1g. School environments**: Proportion of schools that are free from marketing for unhealthy foods and related brands
Labelling (LABEL)	**LABEL1** Application of back of package and front of package labelling systems **1a**. Proportion of all packaged food and drinks that carry a government-endorsed back-of-package or front-of-package labelling system **1b**. Proportion of unhealthy packaged food and drinks carrying back-of-package or front-of-package labelling systems
	**LABEL2** Proportion of packaged food and drinks that are classified as unhealthy but that carry nutrient content claims (e.g., low in sodium’) or health claims (e.g., prevents heart disease’)
Provision (PROV)	PROV1* Proportion of schools in which unhealthy food and drinks (sugary or artificially sweetened drinks, fried foods, salty snacks, sugary snacks) are provided or available for sale regularly^†^ **1a**. Proportion of schools in which **sugary or artificially sweetened drinks**^‡^ are provided or available for sale regularly **1b**. Proportion of schools in which **fried foods**^‡^ are provided or available for sale regularly **1c**. Proportion of schools in which **salty snacks**^‡^ are provided or available for sale regularly **1d**. Proportion of schools in which **sugary snacks**^‡^ (including confectionery) are provided or available for sale regularly
	PROV2* Proportion of schools in which healthy food and drinks are provided or available for sale every day **2a**. Proportion of settings in which **unprocessed or minimally processed fruit** is provided or available for sale every day **2b**. Proportion of settings in which **unprocessed or minimally processed vegetables** (raw or cooked) are provided or available for sale every day **2c**. Proportion of settings with access to **free**, **safe drinking water** at all times
Retail (RET)	**RETAIL1** Proportion of price promotions (e.g., temporary discounts, multi-buys) that relate to unhealthy compared to healthy foods within physical and digital grocery retailers per unit of time
	**RETAIL2** Proportion of healthy checkouts (i.e., display space in and around checkouts that do not have any unhealthy foods or drinks)
	**RETAIL3** Ratio of healthy to unhealthy food outlets (per 100,000 population), by geographic location
Trade (TRA)	TRADE1* Import, production and export of key food categories **1a**. Import volumes by Nova Group and/or food category over a 1 (or 5) year period **1b**. Domestic production volumes by Nova Group and/or food category over a 1 (or 5) year period **1c**. Export volumes by Nova Group and/or food category over a 1 (or 5) year period

*Multiple separate indicators are proposed which can all be drawn from similar data sources and would be reported on as a package.

† Regularly would typically be defined as more than once per week

***‡*** Sugary drinks would likely include soda, 100% fruit juice and fruit drinks, sports drinks and energy drinks; fried foods would likely include foods that have been cooked or heated by deep frying in oil, such as chips, fried chicken or fish; salty snacks would likely include items typically high in sodium including potato chips (crisps) and processed meat snacks; sugary snacks would likely include candy and chocolate, processed baked goods (e.g., muffins), ice cream or frozen treats, and cakes.

#### Composition

The Composition domain considers the nutritional quality of the food supply, comprising both packaged foods and food prepared ‘out-of-home’ (e.g., restaurant/fast-food), including both formal and informal food markets [25]. Four indicators were short-listed in relation to this domain (Table 1). Three indicators focused on packaged foods, and one focused on fast-food chains.

For packaged foods (indicators COMP1, COMP2 and COMP3), the monitoring data underpinning the prioritised indicators would likely be limited to products available in supermarket chains with the largest market share. These indicators rely upon information being available for products (e.g., nutrient declarations and/or ingredients lists), which may differ between countries depending on national labelling regulations and voluntary food industry practices. Across the three indicators related to packaged food, participants recognised the importance of focusing monitoring efforts on a list of priority food categories or developing sampling strategies to minimize the burden of data collection (e.g., regular rolling data collection over time).

The first indicator (COMP1) focuses on the proportion of packaged food and drinks products that are classified as unhealthy. Two potential sub-indicators are proposed, each adopting a different measure related to product healthiness: (a) a national nutrient profiling system; and/or (b) degree of food processing according to Nova. The two different measures of product healthiness were selected to indicate different characteristics of the available products, and in response to feedback from participants (described further below) that indicated that there was no universal agreement on the most appropriate food classification system to use across all indicators, and that simultaneous use of multiple systems would help meet multiple objectives such as national relevance and international comparisons. Participants noted additional value in applying both to understand the nutritional value and degree of processing among products for a comprehensive understanding of the food supply. 

Two indicators (COMP2 and COMP3) focused on specific nutrients in the packaged food supply. COMP2 examines the extent to which products meet relevant government formulation targets for nutrients to limit. Depending on the government targets in a particular country, it may be necessary to have separate indicators for each nutrient (e.g., the proportion of products that meet sodium, sugar and saturated fat targets). Given that relatively few countries have established country-level targets, targets from the WHO [48] for sodium could be useful and applied across countries, and the elimination of trans fat in the food supply provides a benchmark for evaluating levels of trans fat. It was noted that these reformulation targets would likely change over time, which may require consideration in the monitoring of this indicator.

COMP3 examines mean nutrient levels in food and drink products in major healthy and unhealthy food categories. While these are generally and preferably reported per 100mL–100 g, it was noted that the mean level of nutrients could be considered per serve, which is applicable in some country contexts and should be considered if relevant. Across COMP2 and COMP3, when data are available, monitoring of added sugars or free sugars was considered important in addition to total sugars. Participants highlighted that other aspects of food composition could also be considered, including fibre, calcium and non-nutritive sweeteners.

The final indicator, COMP4, related to foods available in major fast food chains. With regards to systems for classifying the healthiness of foods in the ‘out-of-home’ setting, we noted that national nutrient profiling systems specific to this setting are relatively uncommon. The required information to conduct nutrient profiling is not usually publicly available for these foods, and many existing approaches often do not incorporate important elements, such as portion size. We identified that the Health Star Rating has been evaluated for use in restaurant foods [49].

Participants noted that analyses could be disaggregated by company (manufacturer or restaurant chain), which would provide an opportunity to assess individual company progress towards achieving healthier food offerings. Some experts suggested that these data could be reported as sales-weighted to better represent the influence of the food supply on diets; however, others suggested that data required for such analysis would likely be cost prohibitive or unavailable in many countries and settings. It was noted that sampling may need to be adapted in countries where informal markets are larger sources of food acquisition.

#### Price

The Price domain includes the analysis of the price and affordability of individual foods and standardised diets [23]. Three indicators were short-listed, including one in relation to the price of individual foods, one relating to the price of diets, and one relating to the affordability of diets. Sampling (for data collection) was identified as a key element of this indicator, as prices are known to be affected by seasonality, regional differences, systems shocks and price-chain disruptions, as well as price differences between different types of outlets and retailers.

PRICE1 focuses on changes over time in the price of key healthy and unhealthy food and beverages. Given the volatility of food prices and secular trends, participants noted that this would likely need to be reported on a regular (e.g., annual) basis to be most valuable.

PRICE2 describes the cost of different diets. This indicator typically involves integration of product or category-level price estimates with nationally representative dietary intake data to estimate the cost of the current average diet compared to a diet that adheres to national dietary guidelines or a globally accepted healthier dietary pattern. This indicator thus requires regularly collected food price data and dietary intake data upon which an estimate of the current diet can be based.

PRICE3 uses the same methodology as the PRICE2 indicator but presents the estimated cost of the current and recommended diet as a proportion of household income to demonstrate the affordability of diets. Multiple scenarios would be used to describe the affordability of the diet among different population groups, with differing household incomes (households with an income based on minimum wage, households on social assistance, etc.). This approach requires national data on household income and the establishment of a reference household or households (e.g., two adults and two children).

Participants noted the potential for data harvesting methods, such as web-scraping, to support regular price data collection from various regions. Others noted that such methods may not be suitable in all contexts, for example, in countries or regions where online data are not available or there are limited resources for webscraping and associated data cleaning practices. Several participants discussed the potential for using consumer price index (CPI) data that are routinely collected by countries. Participants indicated that, while potentially valuable, there were several limitations to these data [50, 51] which differ between countries and often include a small range of key foods that were not developed with health considerations in mind and are not sampled from all areas. It was noted that international comparisons would require exchange rate parity.

Participants also recognized that data are available on food affordability from the FAO, including a measure on the cost of a healthy diet [50]. The goal of this FAO work is to provide global estimates of food security and monitoring for the Sustainable Development Goals and the eradication of hunger, food insecurity and malnutrition, including the cost of a healthy diet, focusing on regional estimates and changes over time. Some participants indicated that the FAO data are less granular than what is proposed herein, do not compare with the cost of current diets, and may not provide the breadth of information to inform policy at a national level.

#### Promotion

The Promotion domain explores the extent to which children are exposed to the promotion of unhealthy foods [28]. Children are typically exposed to commercial marketing across many media and other settings with which they engage. Participants recognised that it can be challenging to monitor all relevant media/settings, and, thus, monitoring efforts may need to prioritise key media/settings [36, 52]. The proposed strategy puts forth seven media/settings that can be prioritised for monitoring depending on the country context, noting that marketing in retail settings is discussed in the Retail module. Of the seven settings (TV advertising, digital advertising, outdoor advertising, product packaging, community sport-based advertising, sport sponsorship and schools), countries undertaking monitoring were suggested to prioritise at least two for inclusion. For most countries, participants anticipated that digital advertising would be most relevant, as this has been identified in the literature as an increasingly important contributor to children’s advertising exposure [52]. TV advertising was also identified as likely to be of high importance, although trends in children’s engagement with this media should be considered. Participants noted that there may be contexts for which these media are less relevant, such as contexts where few children use digital devices or have internet access, where there are declining rates of commercial TV use, where existing policies are focussed on specific sub-group of children (e.g. aged under 12 years) or where particularly settings, such as sport settings, have important cultural relevance. The selection of media or settings to be monitored may also be based on resources available for data collection and/or existing data on food promotion within the region or neighbouring countries, including industry data on advertising expenditure and others.

Current methods for collecting data include recording television or digital screens for lengthy periods or collecting large volumes of images (e.g. for product packaging marketing), posing challenges with resource intensive data-coding. Participants noted that there were emerging methods whereby data collection and coding methods can be streamlined with web-scraping and machine-learning techniques using image recognition [52], and that additional validation of will support their use in monitoring. Lastly, it was noted that emerging that the best practice approach is to use methods that collect data on ‘actual’ exposures where resource and capacity allows, but ‘potential’ exposure is an alternative.

#### Labelling

The Labelling domain monitors the provision of nutrition information on product labels. LABEL1 includes two sub-indicators relating to the proportion of products that carry nutrition information on the front and the back of package, and the extent to which those products are healthy. Participants noted that monitoring priorities may differ based on whether labelling is mandatory or voluntary. In many countries, some food labelling components are mandatory (e.g., ingredients lists, nutrient declarations, and, in some cases, interpretive front-of-package labelling schemes). In principle, compliance with mandatory labelling is likely to be a lower priority for monitoring efforts than uptake of voluntary measures, although participants noted that implementation of mandatory labelling may vary substantially according to the country context. This indicator requires up-to-date data or images of product packaging to assess the extent of implementation of food labelling, and/or to build the case for strengthening existing measures. Participants noted that this indicator was designed to focus on surveillance of government-endorsed labelling only, and was not intended to include monitoring of industry-led initiatives (such as ‘Guideline Daily Amounts’ labelling or health symbols developed by food companies).

The second labelling indicator (LABEL2) relates to the use of nutrition and health claims on products, and the healthiness of the products carrying claims. Participant feedback highlighted the ability of this indicator to identify the prevalence of potentially misleading uses of nutrition and health claims.

#### Provision

The Provision domain monitors the foods available and the way they are promoted in public sector settings [26]. Input from participants identified that schools were the priority public sector setting for surveillance. As such, the indicators proposed focus on school environments; however, it was noted that these same indicators could be applied to other public sector contexts, such as hospitals, early childhood education centres, correctional facilities, recreation centers, community centres or spaces, libraries and others. The proposed indicators align with a recent report from the UNICEF East Asia and the Pacific Regional Office: the Nutrition Environment Assessment Toolkit for Schools (NEAT-S) [53].

The first provision indicator (PROV1) aims to monitor the proportion of schools that provide or sell unhealthy foods, including foods provided or available for sale in school, nutrition/meal/snack programs and those sold in cafeterias/canteens, school stores and vending machines. It was noted that, when relevant, indicators could be disaggregated by source (school meals/cafeterias/canteens/stores/vending machines) or by the nature of provision (provided for free or sold). The indicator refers to items available ‘regularly’, which would typically be defined as more than once per week. For rotating menus, availability could be assessed on the day of data collection.

The PROV2 indicator examines the proportion of schools that provide or sell healthy foods (including unprocessed or minimally processed fruits and vegetables, and safe drinking water) every day that their food services are operational. Participants noted that a category of whole grain foods may be particularly relevant in some contexts and may require additional criteria for nutrients or other food components in whole grain products. Participants also noted the importance of monitoring school food and nutrition policies, but this was not included in this short-list as policy monitoring was considered out of scope for this work. We also note that monitoring of marketing in schools was included as part of the Promotion domain. Several participants highlighted the importance of monitoring food outlets immediately surrounding schools as these foods may ‘compete’ with school foods, which would fall within the Retail domain [54].

Sampling was identified as a challenge for monitoring in this domain, particularly in countries with multiple sub-national governments and/or large geographic areas. If sampling strategies include a site visit to conduct direct observation, indicators could be adapted for ‘the day of the survey’. Participants also indicated that ethics clearance for in-person data collection in schools can prove a barrier to monitoring efforts, and likely requires governmental support and coordination, although participants noted that some menus may be available online. It was noted that, in some contexts, there are likely to be very few (if any) schools that would have no unhealthy items available; in these contexts, improvements over time would likely be a better indicator than the absolute percentages.

#### Retail

The Retail domain monitors the availability and promotion of foods in retail food environments, encompassing both the spatial distribution of retail outlets in the community as well as in-store environments [24].

The RETAIL1 and RETAIL2 indicators relate to in-store environments. RETAIL1 explores the proportion of price promotions, such as temporary discounts or multi-buys, in relation to unhealthy compared to healthy foods, expressed as an average over time (e.g. per week, per month, etc.). The indicator is designed to capture major grocery retailer chains within a country context, and types of retailers included could be based upon market share data. For example, in many countries, supermarkets would be the primary focus, whereas in some countries monitoring may include convenience stores if they make a prominent contribution to population-level food purchases.

The RETAIL2 indicator relates to the proportion of checkouts in grocery retail settings that are free from unhealthy foods. Challenges with the RETAIL1 and RETAIL2 indicators include sampling strategy, as there is likely to be considerable variation in these indicators over time, as well as between store locations or brands in some instances. While the potential for online data collection exists – and is increasingly relevant as more shoppers turn to digital grocery platforms – this may not be possible in all contexts and may not represent promotions in physical outlets where the majority of grocery shopping still currently takes place. Although there are a range of tools to monitor the healthiness of online grocery retailers under development, large-scale monitoring in these settings will still require advancements in automated data collection to streamline the assessment process.

The RETAIL3 indicator assesses the geographic distribution of retail outlets in the community, and monitors the ratio of different types of food outlets per 100,000 people, by geographic location (such as between states or provinces, level of urbanicity, or area-level socio-economic status). Many participants queried the precision by which healthy and less healthy food outlets can be defined, noting that there are inconsistencies in the literature, and no consensus on methods for doing so, with multiple approaches available [55, 56]. For example, the healthiness and accessibility of some types of outlets (such as supermarkets) may differ between country contexts, and many restaurants offer a wide range of foods that vary in healthiness [13]. Industry-owned data for this indicator are likely to be available for some countries via proprietary data sources, such as the Euromonitor Passport database. Government business registries may also be available to inform this measure in some contexts, and web-scraping approaches to identify different types of food outlets are also emerging [57].

Retail indicators could potentially disaggregate data by geographic location, providing an opportunity to explore elements of inequities between areas of higher and lower socio-economic status, or between regions. RETAIL1 and RETAIL2 could equally be explored by grocery chain, providing understanding of company-level practices. It was also noted that there have been recent advances in government requirements for reporting of sales of healthy and unhealthy food items by food retailers and manufacturers [58], which could be another avenue for monitoring retail settings that requires little data collection effort.

#### Trade & Investment

The Trade and investment domain focuses on monitoring the impacts of international trade and investment liberalization, facilitated by trade and investment agreements, on food environments [29]. The TRADE1 indicator includes three sub indicators for import, production and export of key food categories. It was considered necessary for these three sub indicators to be monitored in tandem to provide an overall portrait of the food supply as linked to global markets. Given the nature of available food trade data that is typically classified at a high level of aggregation, the indicators in this domain are recommended to be assessed according to the level of processing, using Nova. Participants recommended that monitoring should assess time trends (for example, five year periods), and likely could be monitored retrospectively (as far back as 1990).

Participants highlighted the importance of investigating foreign direct investment into the food system. However, determining potential health-oriented indicators using the available data was identified a challenge for monitoring this area, as the data are generally not granular enough to examine food-specific trends.

### Prioritisation of Key Indicators

The results from the prioritisation exercise within the online survey are shown in Online Resource 2, which shows how often indicators were selected within the top seven, and the distribution of the assigned rankings (from 1 to 7) for each indicator. The final set of prioritised indicators are presented in Box 2.

**Box 2**. Prioritised indicators for monitoring and assessment of the healthiness of food environments at the country level.

**Table:** 

• COMPOSITION (COMP1): Proportion of packaged food and drinks products that are classified as unhealthy according to a nationally relevant nutrient profiling system and/or the Nova system
• PROMOTION (PROMO): Frequency of exposures to unhealthy food advertisements in 2 primary media/settings to monitor from television, digital, outdoor, elite sport, community sport, product packaging, and school environments.
• PRICE (PRICE3): Price of a healthy diet compared to the price of a current diet as a proportion of income
• PROVISION (PROV1): Proportion of schools in which unhealthy foods (sugary or artificially sweetened drinks, fried foods, salty snacks, sugary snacks) are provided or available for sale regularly
• LABELLING (LABEL1): Application of back of package and front of package labelling systems

### Cross-Cutting Implementation Considerations

#### Classifying the Healthiness of Foods

Most of the proposed indicators necessitate the categorical classification of foods as ‘healthy’ or ‘unhealthy’. Initial reflections as well as feedback from the broader group highlighted the lack of consensus regarding food classification systems that could be appropriately applied across settings [44–46].

Reflections from participants indicated the importance of applying a country-specific food classification system, ideally developed or endorsed by governments, when available, to enhance policy relevance in a local context. These systems are typically designed to align with national nutrition guidelines or policy objectives (e.g., Health Canada’s front-of-package labelling regulations [59], Food Standards Australia New Zealand Nutrient Profiling Scoring Criterion [60]). WHO regional nutrient profiling models [61–65] could also be used. It was noted, however, that application of country-specific tools or systems may not facilitate international comparisons.

Participants noted the potential value in applying the Nova classification system as a measure of the level of food processing for some of the selected indicators [66]. The Nova system was identified as particularly useful for some indicators (e.g., in relation to trade and investment) where ingredients or nutrient-based data are not available.

Feedback suggested the inclusion of a model that incorporates both nutrients and level of processing; however, there is still a limited number of existing classification systems that have incorporated both elements that would be suitable across country contexts [67]. In consideration of this feedback, we did not recommend the use of a single scheme across the set of prioritised indicators. For some indicators we proposed use of multiple different systems (e.g., refer to COMP1, COMP4), and for others we selected the system that was most fit-for-purpose (e.g., TRADE1). Overall, we think this balances the need for policy relevance, whilst facilitating cross country comparisons where possible. Development of a harmonized food profiling approach remains a priority.

Feedback also indicated the potential utility of algorithmic food classification schemes that provide an indicator of overall nutrition quality of foods, either across the board or within food categories. Prominent examples of such schemes include the Health Star Rating and Nutri-Score, although there was no consensus on which may be most appropriate, or if other systems may be preferred. The Health Star Rating system has been previously used for international comparative purposes in food environments [68, 69]; however, participants expressed that other systems could be used for international comparison purposes and that the application of such a system may not be policy relevant in some contexts.

#### Key Food Categories

For many of the policy domains examined, sampling (for data collection) and reporting of indicators relies on the inclusion of specific food categories (e.g., sugary drinks, breakfast cereals, etc.) considered most relevant for monitoring food environments. The selection of priority food products may differ depending on country context (e.g., dietary patterns, food culture, and local policy priorities) but likely fall into similar general groups. The identification of specific food categories for international monitoring was beyond the scope of this paper, although participants proposed considerations for identifying such categories. Suggestions included: food categories that are the biggest contributors to sodium, sugar or saturated/trans fat in national diets; food categories that make the largest contribution to the total dietary pattern; and/or food categories that align with national or international nutrition-related policies or strategies [70].

#### Benchmarks

Results suggested that there was generally strong support to apply benchmarking criteria to assess and communicate the extent to which the results from food environment monitoring indicate favorable or unfavorable conditions from a health perspective. For example, in Australia’s Food Environment Dashboard [18], indicators were assessed against set criteria to assist with the interpretation of data with ‘green’ used to indicate where food environments were likely to be promoting health, ‘amber’ used to indicate areas needing further improvement to promote health, and ‘red’ used to indicate aspects that were unhealthy/not supportive of health. In addition, there was support for benchmarks to be used to indicate trends over time (e.g., improving, unchanged, worsening). Benchmarks for each of the indicators were not proposed at this stage.

## Discussion

This paper proposed a set of prioritised indicators for monitoring and assessment of the healthiness of food environments at the country level. The short-listed set of indicators included 16 indicators (some of which incorporate sub-indicators) across seven domains including food composition, prices, marketing, labelling, retail, provision in public sector settings, trade and investment. We also identified five indicators to prioritise as part of large-scale surveillance and monitoring: the proportion of packaged food and drinks products that are classified as unhealthy; the frequency of exposures to unhealthy food promotion in two primary media/settings; the price of a healthy diet compared to the price of the current diet as a proportion of income; the proportion of schools in which unhealthy foods are provided or available for sale regularly; and uptake of back of package and front of package food labelling. Critically, the selection and application of particular indicators is likely to require adaptation to the local context, including modification based on the resources available, the country-level culture and policy setting as well as the food system structure within which the food environment operates.

The prioritised indicators represent synergistic elements of the food environment, and potential data collection strategies could inform multiple indicators. Integrated sampling strategies that leverage data collection for multiple indicators could help establish a strategically coherent and feasible monitoring system that minimizes demands on financial, human and technical resources. For example, data collection for the nutritional quality of the food supply could measure the healthiness of foods that are available and marketed in schools or supermarkets, could collect images of products to examine labelling practices and could incorporate collection of price data.

The study findings must be considered in the context of resource-constrained environments. Globally, there are major surveillance gaps related to dietary intake and health-related behaviours, which make monitoring of key targets such as the Sustainable Development Goals increasingly difficult. This study aimed to help decision-makers identify key surveillance areas that are likely to be feasible to collect and can inform both national and international policy priorities. Nonetheless, implementing these recommendations will require government commitment to collect and house food environment data, as well as expertise in procuring, cleaning, analysing and interpreting the data. The proposed indicators could be adapted or tailored, based on available resources, existing monitoring systems, application in a country context, and data collection approaches used. Importantly, the set of five prioritised indicators may not all be relevant in some contexts, and may be supplemented or replaced by additional indicators (e.g., from the 16-item short-list) where deemed appropriate. For example, while there were no indicators from the Retail domain selected as part of the set of five priority indicators, nor indicators related to restaurant and fast food settings, these remain critical elements of food environments in many settings and are increasingly central to government approaches to improve food environments [71, 72], and thus may be important monitoring elements to consider.

This study points to burgeoning methodological approaches to evaluate food environments in relation to efforts to improve population diets and prevent NCDs. The current literature providing data on food environments is comprised largely of researcher-driven studies that are typically dependent on ad hoc grant funding and do not generate consistent and comparable data that can be used for monitoring over time [7]. Research methods and their ensuing results are often tailored to a particular research, policy question or context, leading to an increasing number of outcomes reported. Variability in research approaches as well as reporting of outcomes and indicators related to the healthiness of food environments reduces the ability to collate results and compare across countries [11] points to the need for institutionalized, government-led efforts to ensure that data are available to inform policy decisions. This study may help researchers identify key outcomes to report within their respective research programs, supporting efforts to increase global data harmonization. This is critical for enabling cross-country comparisons, tracking progress toward global nutrition targets, and informing coordinated policy responses. Consideration should be given to the existing national or subnational monitoring systems that are already in place [73], which may support the scalability and sustainability of these efforts.

We acknowledge that the methods for monitoring and assessing the healthiness of food environments are rapidly evolving, with advances in data collection technology and artificial intelligence that are likely to shape the resources required to collect and analyse these data. Importantly, the study highlights a lack of consensus among experts in key areas of food environments research, including food classification systems to classify the healthiness of foods and food outlets, which underpins policy decisions. This reflects ongoing siloed approaches to healthier food environments and greater transdisciplinary and international efforts to further global coherence, as the lack of a consensus may stall or undermine policy action.

### Strengths and Limitations

A key strength of this study is that we brought together indicators and related findings from a large volume of disparate studies, using a definition of food environments and conceptual framework that have been well established [3]. The study was highly consultative, drawing upon the broad expertise of an international research network, from diverse geographic regions, to inform the current findings [6].

The study has notable limitations. While the initial narrative review was conducted only in English and French, the global research team and international network reduce the likelihood that key indicators were overlooked in subsequent steps. Of note, evidence from methodological studies suggests that most indexed scientific literature is published in English, and that excluding non-English languages typically affects a relatively small proportion of studies and rarely changes overall review conclusions [74]. The indicators developed are based largely upon a literature evaluating food environments in high or upper-middle income countries that are already well into the nutrition transition [75]. We acknowledge that the focus of some of the prioritised indicators have lesser relevance in the current context of some LMICs, where traditional markets or informal vendors remain dominant [10]. As food environments change globally to become increasingly industrialized [12, 13, 75–77] and dominated by chain retailers [13], the establishment of monitoring systems is essential to track the speed of change, and to ensure that the negative impacts of commercialized food systems on NCD trajectories are mitigated as fully as possible. Furthermore, the feasibility of some of the recommended monitoring strategies rely on supportive government policies that are not universally implemented. For example, in areas where nutritional information is not mandated on packages, there are barriers to food supply monitoring that would reduce the feasibility of adopting the proposed priority indicators. Importantly, this review did not include indicators for monitoring public or private sector policies, which are encompassed in other INFORMAS work [20, 21].

The current analysis did not incorporate indicators for breast-milk substitutes, complementary foods or follow-on formulas. Given the identified lack of adherence to the International Code of Marketing of Breast-milk Substitutes by many companies [78, 79], indicators related to breast-milk substitute marketing, labelling, prices and retailing practices, as well as documentation of the number of companies that are violating the Code, may be additional indicators to consider [80]. Similarly, specific attention to the marketing practices for infant and toddler foods (including complementary foods and follow-on formulas) may deserve special attention in some cases.

The current approach, guided by the INFORMAS framework, did not incorporate environmental sustainability, nor did it explicitly incorporate social justice and equity into all indicators. These are two limitations that are relevant to food environments and public health more broadly. While equity was not proposed as an initial consideration for each indicator, this criterion was sometimes explicit in the proposed sampling strategies or in indicators that are particularly sensitive to equity, such as indicators in the price domain. A criterion with a focus equity or importance among vulnerable populations could be considered in future prioritisation work.

We also did not provide specific lists of key food categories that should be considered for monitoring, which may be useful in the future to inform monitoring efforts. Future work may include broader considerations of environmental sustainability, equity and undernutrition, which are globally recognized as intertwined and concomitant factors that influence human and planetary health [81, 82]. The next step for this research is to establish evidence-informed benchmarks to further support the monitoring and accountability of all policy actors to establish healthier food environments for all [83].

## Conclusion

Most countries lack comprehensive surveillance tools to monitor and assess the healthiness of food environments, and access to quality food environment data remains limited, in part due to a lack of investment in robust information infrastructure. The lack of focus on monitoring food environments is problematic given that food environments are a major driver of obesity and diet-related diseases, which are a substantial burden on health and healthcare costs globally. This paper provides a framework that can contribute to more standardized, global indicators for the monitoring and assessment of the healthiness of food environments. The study may also help inform the development of institutionalized, government-led routine monitoring to address the increasing burden of NCDs. Coherent, resource-sensitive monitoring can be used to inform key policy decisions and drive increased policy action to support healthier food environments globally.

## Key References

- Kelly B, Backholer K, Boyland E, Kent MP, Bragg MA, Karupaiah T et al. Contemporary Approaches for Monitoring Food Marketing to Children to Progress Policy Actions. Current Nutrition Reports. 2023;12(1):14–25. 10.1007/s13668-023-00450-7.
  - ○ Summarizes essential monitoring approaches for food marketing to children in various policy environments, and describes the necessary data to support policy implementation and evaluation.
- Scapin T, Romaniuk H, Feeley A, Corrêa KP, Kupka R, Gomez-Donoso C et al. Global food retail environments are increasingly dominated by large chains and linked to the rising prevalence of obesity. Nature Food. 2025;6(3):283-95. 10.1038/s43016-025-01134-x.
  - ○ Demonstrates that retail food environments are increasingly shifting towards environments controlled by large chain food retailers with increasing digital sales, which were positively correlated with rates of obesity. These global shifts demonstrate the need for monitoring to inform policy decisions to ensure that such environments promote health and reduce risk of obesity and other noncommunicable disease.
- Popkin BM, Miles DR, Taillie LS, Dunford EK. A policy approach to identifying food and beverage products that are ultra-processed and high in added salt, sugar and saturated fat in the United States: a cross-sectional analysis of packaged foods. The Lancet Regional Health - Americas. 2024;32:100713. 10.1016/j.lana.2024.100713.
  - ○ Describes one new food profiling approach that combined nutrient levels with indicators of ultra-processed foods. In our study, a combined approach using both nutrients and levels of processing was highlighted as essential in working towards harmonization of food environment monitoring data.
- Backholer K, Gupta A, Zorbas C, Bennett R, Huse O, Chung A et al. Differential exposure to, and potential impact of, unhealthy advertising to children by socio‐economic and ethnic groups: A systematic review of the evidence. Obesity Reviews. 2021 Mar;22(3):e13144.
  - ○ Demonstrates inequities in exposure to key components of food environments and the need for monitoring to ensure that inequities are not further exacerbated.
- Fanzo J, Haddad L, McLaren R, Marshall Q, Davis C, Herforth A et al. The Food Systems Dashboard is a new tool to inform better food policy. Nature Food. 2020;1(5):243-6
  - ○ Describes the importance of good global food environments data to feed monitoring efforts and policy action, and underscores the lack of global food environments data currently available.

## Supplementary Information

Below is the link to the electronic supplementary material.

Supplementary Material 1 (DOCX 60.2 KB)

Supplementary Material 2 (DOCX 26.0 KB)

## Data Availability

Data will be made available as necessary upon reasonable request.

## References

[1] Afshin A, Sur PJ, Fay KA, Cornaby L, Ferrara G, Salama JS, et al. Health effects of dietary risks in 195 countries, 1990–2017: a systematic analysis for the Global Burden of Disease Study 2017. Lancet. 2019;393(10184):1958–72. 30954305 10.1016/S0140-6736(19)30041-8 PMC6899507

[2] Swinburn B, Egger G, Raza F. Dissecting obesogenic environments: the development and application of a framework for identifying and prioritizing environmental interventions for obesity. Prev Med. 1999;29(6):563. 10600438 10.1006/pmed.1999.0585

[3] Swinburn B, Sacks G, Vandevijvere S, Kumanyika S, Lobstein T, Neal B, et al. INFORMAS (International Network for Food and Obesity/non-communicable diseases Research, Monitoring and Action Support): overview and key principles. Obes Rev. 2013;14(S1):1. 24074206 10.1111/obr.12087

[4] World Health Organization. WHO acceleration plan to stop obesity. 2023. https://www.who.int/publications/i/item/9789240075634

[5] UN General Assembly. Political declaration of the high-level meeting of the general assembly on the prevention and control of non-communicable diseases. New York: UN; 2011.

[6] INFORMAS. INFORMAS. Benchmarking food environments. 2018. http://www.informas.org/

[7] Sacks G, Kwon J, Vandevijvere S, Swinburn B. Benchmarking as a public health strategy for creating healthy food environments: an evaluation of the INFORMAS initiative (2012–2020). Annu Rev Public Health. 2021;42:345–62. 33351647 10.1146/annurev-publhealth-100919-114442

[8] Kelly B, Vandevijvere S, Ng S, Adams J, Allemandi L, Bahena-Espina L, et al. Global benchmarking of children’s exposure to television advertising of unhealthy foods and beverages across 22 countries. Obes Rev. 2019;20:116–28. 30977265 10.1111/obr.12840 PMC6988129

[9] Turner C, Aggarwal A, Walls H, Herforth A, Drewnowski A, Coates J, et al. Concepts and critical perspectives for food environment research: a global framework with implications for action in low-and middle-income countries. Global food Secur. 2018;18:93–101.

[10] Downs SM, Ahmed S, Fanzo J, Herforth A. Food environment typology: advancing an expanded definition, framework, and methodological approach for improved characterization of wild, cultivated, and built food environments toward sustainable diets. Foods. 2020;9(4):532. 10.3390/foods9040532. 32331424 10.3390/foods9040532 PMC7230632

[11] Sacks G, Robinson E, Cameron AJ. Issues in measuring the healthiness of food environments and interpreting relationships with diet, obesity and related health outcomes. Curr Obes Rep. 2019;8(2):98–111. 10.1007/s13679-019-00342-4. 30879246 10.1007/s13679-019-00342-4

[12] Popkin BM, Corvalan C, Grummer-Strawn LM. Dynamics of the double burden of malnutrition and the changing nutrition reality. Lancet. 2020;395(10217):65–74. 10.1016/s0140-6736(19)32497-3. 31852602 10.1016/S0140-6736(19)32497-3 PMC7179702

[13] Scapin T, Romaniuk H, Feeley A, Corrêa KP, Kupka R, Gomez-Donoso C, et al. Global food retail environments are increasingly dominated by large chains and linked to the rising prevalence of obesity. Nat Food. 2025;6(3):283–95. 10.1038/s43016-025-01134-x. 40033144 10.1038/s43016-025-01134-x PMC11932928

[14] Rabiei R, Bastani P, Ahmadi H, Dehghan S, Almasi S. Developing public health surveillance dashboards: a scoping review on the design principles. BMC Public Health. 2024;24(1):392. 10.1186/s12889-024-17841-2. 38321469 10.1186/s12889-024-17841-2 PMC10848508

[15] Fanzo J, Haddad L, McLaren R, Marshall Q, Davis C, Herforth A, et al. The food systems dashboard is a new tool to inform better food policy. Nat Food. 2020;1(5):243–6.

[16] The Global Alliance for Improved Nutrition (GAIN). TCCS, and Cornell University College of Agriculture and Life Sciences. Food Systems Dashboard. 2025.

[17] Tóth K, Ács S, Aschberger K, Barbero Vignola G, Bopp S, Caivano A, et al. EU food system monitoring framework – From concepts to indicators. Publications Office of the European Union. 2024.

[18] Deakin University. Australia’s Food Enviroment Dashboard. 2021. https://foodenvironmentdashboard.com.au/

[19] University of Auckland. Aotearoa’s Food Environment Dashboard. n.d. https://foodenvironmentsaotearoa.nz/

[20] Swinburn B, Vandevijvere S, Kraak V, Sacks G, Snowdon W, Hawkes C, et al. Monitoring and benchmarking government policies and actions to improve the healthiness of food environments: a proposed Government Healthy Food Environment Policy Index. Obes Rev. 2013;14(S1):24. 24074208 10.1111/obr.12073

[21] Sacks G, Swinburn B, Kraak V, Downs S, Walker C, Barquera S, et al. A proposed approach to monitor private-sector policies and practices related to food environments, obesity and non-communicable disease prevention. Obes Rev. 2013;14(S1):38. 24074209 10.1111/obr.12074

[22] Vandevijvere S, Monteiro C, Krebs-Smith S, Lee A, Swinburn B, Kelly B, et al. Monitoring and benchmarking population diet quality globally: a step‐wise approach. Obes Rev. 2013;14:135–49. 24074217 10.1111/obr.12082

[23] Lee A, Mhurchu CN, Sacks G, Swinburn B, Snowdon W, Vandevijvere S, et al. Monitoring the price and affordability of foods and diets globally. Obes Rev. 2013;14:82–95. 24074213 10.1111/obr.12078

[24] Ni Mhurchu C, Vandevijvere S, Waterlander W, Thornton LE, Kelly B, Cameron AJ, et al. <article-title update="added">Monitoring the availability of healthy and unhealthy foods and non‐alcoholic beverages in community and consumer retail food environments globally. Obes Rev. 2013;14:108–19. 24074215 10.1111/obr.12080

[25] Neal B, Sacks G, Swinburn B, Vandevijvere S, Dunford E, Snowdon W, et al. Monitoring the levels of important nutrients in the food supply. Obes Rev. 2013;14(S1):49–58. 24074210 10.1111/obr.12075

[26] L’Abbé M, Schermel A, Minaker L, Kelly B, Lee A, Vandevijvere S, et al. Monitoring foods and beverages provided and sold in public sector settings. Obes Rev. 2013;14:96–107. 24074214 10.1111/obr.12079

[27] Rayner M, Wood A, Lawrence M, Mhurchu CN, Albert J, Barquera S, et al. Monitoring the health-related labelling of foods and non‐alcoholic beverages in retail settings. Obes Rev. 2013;14:70–81. 24074212 10.1111/obr.12077

[28] Kelly B, King L, Baur L, Rayner M, Lobstein T, Monteiro C, et al. Monitoring food and non-alcoholic beverage promotions to children. Obes Rev. 2013;14:59–69. 24074211 10.1111/obr.12076

[29] Friel S, Hattersley L, Snowdon W, Thow AM, Lobstein T, Sanders D, et al. Monitoring the impacts of trade agreements on food environments. Obes Rev. 2013;14:120–34. 24074216 10.1111/obr.12081

[30] Vandevijvere S, Mackay S, D’Souza E, Swinburn B. The first INFORMAS national food environments and policies survey in New Zealand: a blueprint country profile for measuring progress on creating healthy food environments. Obes Rev. 2019;20(S2):141–60. 10.1111/obr.12850. 31483561 10.1111/obr.12850

[31] Ni Mhurchu CINFORMAS, Protocol. Food Retail – Food availability in supermarkets FULL – v1.1. 2017. https://cdn.auckland.ac.nz/assets/fmhs/soph/globalhealth/informas/docs/Retail%20Instore%20Food%20Availability%20%20Protocol%20SAMPLE-v1docx.pdf

[32] Kelly B. INFORMAS protocol: Promotion – outdoor advertising (school zones). 2017. https://figshare.com/articles/INFORMAS_protocol_Outdoor_advertising_school_zones_/5701102

[33] Lee A. INFORMAS protocol: Food prices module. 2017. https://figshare.com/articles/INFORMAS_Protocol_Food_Prices_Module/5627440

[34] Vandevijvere S, Mackay S, D’Souza E, Swinburn. B. How healthy are New Zealand food environments? A comprehensive assessment 2014-20172018.

[35] Rayner M, Vandevijvere SINFORMAS, Protocol. Food Label Module. 2017. 10.17608/k6.auckland.5673643.v1.

[36] Potvin Kent M, Mulligan C, Pauzé E, Pinto A, Remedios L. The food and beverage marketing monitoring framework for Canada: development, implementation, and gaps. Food Policy. 2024;122:102587. 10.1016/j.foodpol.2023.102587.

[37] Olstad DL, Prowse RJL, Raine KD, Tomlin D, Kirk SF, McIsaac J-L, et al. Baseline results from the eat, play, live trial: a randomized controlled trial within a natural experiment examining the role of nutrition policy and capacity building in improving food environments in recreation and sport facilities. Food Policy. 2020;92:101870. 10.1016/j.foodpol.2020.101870. 10.1186/s12966-019-0811-8 PMC6593504 31238919

[38] Garnica Rosas L, Mensink GBM, Finger JD, Schienkiewitz A, Do S, Wolters M, et al. Selection of key indicators for European policy monitoring and surveillance for dietary behaviour, physical activity and sedentary behaviour. Int J Behav Nutr Phys Act. 2021;18(1):48. 10.1186/s12966-021-01111-0. 33794923 10.1186/s12966-021-01111-0 PMC8015190

[39] Backholer K, Gupta A, Zorbas C, Bennett R, Huse O, Chung A, et al. Differential exposure to, and potential impact of, unhealthy advertising to children by socio-economic and ethnic groups: a systematic review of the evidence. Obes Rev. 2020. 10.1111/obr.13144. 33073488 10.1111/obr.13144

[40] Zorbas C, Eyles H, Orellana L, Peeters A, Mhurchu CN, Riesenberg D, et al. Do purchases of price promoted and generic branded foods and beverages vary according to food category and income level? Evidence from a consumer research panel. Appetite. 2020;144:104481. 10.1016/j.appet.2019.104481. 31589906 10.1016/j.appet.2019.104481

[41] The Food Monitoring Group. International collaborative project to compare and track the nutritional composition of fast foods. BMC Public Health. 2012;12(1):559. 10.1186/1471-2458-12-559. 22838731 10.1186/1471-2458-12-559 PMC3490731

[42] Riesenberg D, Backholer K, Zorbas C, Sacks G, Paix A, Marshall J, et al. Price promotions by food category and product healthiness in an Australian supermarket chain, 2017–2018. Am J Public Health. 2019;109(10):1434–9. 10.2105/ajph.2019.305229. 31415196 10.2105/AJPH.2019.305229 PMC6727276

[43] Kent MP, Velazquez CE, Pauzé E, Cheng-Boivin O, Berfeld N. Food and beverage marketing in primary and secondary schools in Canada. BMC Public Health. 2019;19(1):114. 30691422 10.1186/s12889-019-6441-x PMC6348619

[44] World Health Organization. Use of nutrient profile models for nutrition and health policies: meeting report on the use of nutrient profile models in the WHO European Region, September 2021. World Health Organization. Regional Office for Europe. 2022.

[45] Martin C, Turcotte M, Cauchon J, Lachance A, Pomerleau S, Provencher V, et al. Systematic review of nutrient profile models developed for nutrition-related policies and regulations aimed at noncommunicable disease prevention–an update. Adv Nutr. 2023. 10.1016/j.advnut.2023.08.013. 37659696 10.1016/j.advnut.2023.08.013 PMC10721541

[46] Astrup A, Monteiro CA. Does the concept of ultra-processed foods help inform dietary guidelines, beyond conventional classification systems? Debate consensus. Am J Clin Nutr. 2022;116(6):1489–91. 10.1093/ajcn/nqac230. 36253965 10.1093/ajcn/nqac230

[47] Commonwealth of Australia. Health Star Rat Syst. 2016. https://www.healthstarrating.gov.au/

[48] World Health Organization. WHO global sodium benchmarks for different food categories. World Health Organization. 2024.

[49] Dunford EK, Wu JH, Wellard-Cole L, Watson W, Crino M, Petersen K, et al. A comparison of the Health Star Rating system when used for restaurant fast foods and packaged foods. Appetite. 2017;117:1–8. 28603059 10.1016/j.appet.2017.06.005

[50] FAO I, UNICEF, WFP and WHO. The State of Food Security and Nutrition in the World 2024 – Financing to end hunger, food insecurity and malnutrition in all its forms. Rome2024.

[51] Luongo G, Tarasuk V, Cahill LE, Hajizadeh M, Yi Y, Mah CL. Cost of a healthy diet: a population-representative comparison of 3 diet cost methods in Canada. J Nutr. 2024;154(11):3424–36. 10.1016/j.tjnut.2024.09.002. 39270849 10.1016/j.tjnut.2024.09.002 PMC11600118

[52] Kelly B, Backholer K, Boyland E, Kent MP, Bragg MA, Karupaiah T, et al. Contemporary approaches for monitoring food marketing to children to progress policy actions. Curr Nutr Rep. 2023;12(1):14–25. 10.1007/s13668-023-00450-7. 36746878 10.1007/s13668-023-00450-7 PMC9974707

[53] UNICEF East Asia and the Pacific Regional Office. Nutrition Environment Assessment Toolkit for Schools (NEAT-S) for East Asia and Pacific. Bangkok: UNICEF; 2024.

[54] Davis B, Pechmann C. When students patronize fast-food restaurants near school: the effects of identification with the student community, social activity spaces and social liability interventions. Int J Environ Res Public Health. 2023. 10.3390/ijerph20054511. 36901521 10.3390/ijerph20054511 PMC10002251

[55] Pulker CE, Trapp GSA, Fallows M, Hooper P, McKee H, Pollard CM. Food outlets dietary risk (FODR) assessment tool: study protocol for assessing the public health nutrition risks of community food environments. Nutr J. 2020;19(1):122. 10.1186/s12937-020-00641-w. 33183279 10.1186/s12937-020-00641-w PMC7663896

[56] Moayyed H, Kelly B, Feng X, Flood V. Evaluation of a ‘healthiness’ rating system for food outlet types in Australian residential communities. Nutr Diet. 2017;74(1):29–35. 10.1111/1747-0080.12286. 28731554 10.1111/1747-0080.12286

[57] Jia SS, Luo X, Gibson AA, Partridge SR. Developing the DIGIFOOD dashboard to monitor the digitalization of local food environments: interdisciplinary approach. JMIR Public Health Surveill. 2024;10(1):e59924. 39137032 10.2196/59924 PMC11350305

[58] UK Government. Healthy food revolution to tackle obesity epidemic. 2025. https://www.gov.uk/government/news/healthy-food-revolution-to-tackle-obesity-epidemic

[59] Health Canada. Front-of-package nutrition symbol labelling guide for industry (Version 2). 2023. https://www.canada.ca/en/health-canada/services/food-nutrition/legislation-guidelines/guidance-documents/front-package-nutrition-symbol-labelling-industry.html#a5.2

[60] Food Standards Australia and New Zealand. Short guide for industry to the NPSC. 2022. https://www.foodstandards.gov.au/business/labelling/Short-guide-for-industry-to-the-NPSC

[61] World Health Organization Europe. WHO Regional Office for Europe nutrient profile model: second edition. 2023. https://iris.who.int/server/api/core/bitstreams/cbaf0eb1-ea08-4999-a959-d016f0f0db6f/content

[62] Organization WH. Nutrient Profile Model for the WHO African Region: a tool for implementing WHO recommendations on the marketing of foods and non-alcoholic beverages to children. Nutrient Profile Model for the WHO African Region: a tool for implementing WHO recommendations on the marketing of foods and non-alcoholic beverages to children. 2019.

[63] World Health Organization Regional Office of South-East Asia. WHO nutrient profile model for South-East Asia Region. 2017. https://iris.who.int/server/api/core/bitstreams/f14a9ed9-0bcc-49cb-923d-e071f63d3297/content

[64] Pan American Health Organization. Pan American Health Organization Nutrient Profile Model. 2016. https://iris.paho.org/bitstream/handle/10665.2/18621/9789275118733_eng.pdf

[65] World Health Organization Regional Office for the Western Pacific. WHO Nutrient Profile Model for the Western Pacific Region: A tool to protect children from food marketing. 2016. https://iris.who.int/server/api/core/bitstreams/841c0283-2454-4512-92ea-82cc3f7a2a39/content

[66] Monteiro CA, Cannon G, Levy RB, Moubarac J-C, Louzada ML, Rauber F, et al. Ultra-processed foods: what they are and how to identify them. Public Health Nutr. 2019;22(5):936–41. 30744710 10.1017/S1368980018003762 PMC10260459

[67] Popkin BM, Miles DR, Taillie LS, Dunford EK. A policy approach to identifying food and beverage products that are ultra-processed and high in added salt, sugar and saturated fat in the United States: a cross-sectional analysis of packaged foods. The Lancet Regional Health - Americas. 2024;32:100713. 10.1016/j.lana.2024.100713. 38495314 10.1016/j.lana.2024.100713 PMC10943474

[68] Dunford EK, Ni Mhurchu C, Huang L, Vandevijvere S, Swinburn B, Pravst I, et al. A comparison of the healthiness of packaged foods and beverages from 12 countries using the Health Star Rating nutrient profiling system, 2013–2018. Obes Rev. 2019;20:107–15. 31328385 10.1111/obr.12879

[69] Access to Nutrition initiative. Global Index 2024. 2024. https://accesstonutrition.org/index/global-access-to-nutrition-index/

[70] Vanderlee LV, McLaughlin C, Olstad A, Ahmed DL, Kirk M, Labonté S, Mah MÈ, Minaker CL, Moubarac L, Mulligan JC, Potvin C, Kent M, Provencher V, Prowse R, Raine K, Schram A, Vergeer L, L’Abbé. MR. An in-depth look at Canada’s food environments: Results from INFORMAS Canada. Québec2025 Contract No.: ISBN 978-2-9822281-3-9 (PDF).

[71] Blueprint by Nesta. Blueprint for halving obesity: Policy brief. n.d.

[72] UK Government. Restricting promotions of products high in fat, sugar or salt by location and by volume price. 2023. https://www.gov.uk/government/publications/restricting-promotions-of-products-high-in-fat-sugar-or-salt-by-location-and-by-volume-price

[73] United Nations Children’s Fund (UNICEF). Strengthening Nutrition in Schools in Sri Lanka. Kathmandu2025.

[74] Morrison A, Polisena J, Husereau D, Moulton K, Clark M, Fiander M, et al. The effect of English-language restriction on systematic review-based meta-analyses: a systematic review of empirical studies. Int J Technol Assess Health Care. 2012;28(2):138–44. 10.1017/s0266462312000086. 22559755 10.1017/S0266462312000086

[75] Popkin BM, Adair LS, Ng SW. Global nutrition transition and the pandemic of obesity in developing countries. Nutr Rev. 2012;70(1):3–21. 10.1111/j.1753-4887.2011.00456.x. 22221213 10.1111/j.1753-4887.2011.00456.x PMC3257829

[76] Popkin BM, Hawkes C. Sweetening of the global diet, particularly beverages: patterns, trends, and policy responses. Lancet Diabetes Endocrinol. 2016;4(2):174–86. 10.1016/s2213-8587(15)00419-2. 26654575 10.1016/S2213-8587(15)00419-2 PMC4733620

[77] Popkin BM, Reardon T. Obesity and the food system transformation in Latin America. Obes Rev. 2018;19(8):1028–64. 10.1111/obr.12694. 29691969 10.1111/obr.12694 PMC6103889

[78] Organization WH. International code of marketing of breast-milk substitutes. World Health Organization. 1981.

[79] Becker GE, Zambrano P, Ching C, Cashin J, Burns A, Policarpo E, et al. Global evidence of persistent violations of the international code of marketing of breast-milk substitutes: a systematic scoping review. Maternal & Child Nutrition. 2022;18:e13335. 35313063 10.1111/mcn.13335 PMC9113471

[80] & WHO, UNICEF. NETCODE toolkit monitoring the marketing of breast-milk substitutes: Protocol for periodic assessments. In: World Health Organization. Geneva, Switzerland. 2017.

[81] Swinburn BA, Kraak VI, Allender S, Atkins VJ, Baker PI, Bogard JR, et al. The global syndemic of obesity, undernutrition, and climate change: The Lancet Commission report. Lancet. 2019;393(10173):791–846. 30700377 10.1016/S0140-6736(18)32822-8

[82] Burgaz C, Van-Dam I, Garton K, Swinburn BA, Sacks G, Asiki G, et al. Which government policies to create sustainable food systems have the potential to simultaneously address undernutrition, obesity and environmental sustainability? Globalization health. 2024;20(1):56. 39068420 10.1186/s12992-024-01060-w PMC11282665

[83] Swinburn B, Kraak V, Rutter H, Vandevijvere S, Lobstein T, Sacks G, et al. Strengthening of accountability systems to create healthy food environments and reduce global obesity. Lancet. 2015;385(9986):2534–45. 25703108 10.1016/S0140-6736(14)61747-5

