# Supplementary material for: Prioritising Indicators for Large-scale Monitoring and Assessment of Food Environments for Public Health
Source: Curr Obes Rep. 2026 Apr 22;15(1):37. doi: 10.1007/s13679-026-00705-8 (PMC13102733; doi:10.1007/s13679-026-00705-8)
Supplement: Supplementary file 1 — Supplementary Material 1 (DOCX 60.2 KB) [file 13679_2026_705_MOESM1_ESM.docx]

Corresponding author: Lana Vanderlee (lana.vanderlee@fsaa.ulaval.ca)

**Table 1.** Complete food environment indicators identified in the literature assessing and monitoring food environments for public health, categorized by INFORMAS policy domain

**FOOD COMPOSITION**

| **NATIONAL FOOD SUPPLY** | |
| --- | --- |
| **1** | Amounts of fruits and vegetables available in the food supply (g/person/day) |
| **2** | Amount of kilocalories/kilojoules per person per day available in the food supply |
| **3** | Growth in retail value of ultra-processed food sales, over a 1 (or 5) year (in %) |
| **4** | Retail value of ultra-processed food sales (in USD million) |
| **5** | Nutrition Functional Diversity index score |
| **6** | Retail value of ultra-processed food sales per person (in USD/capita) |
| **PACKAGED FOODS** | |
| **7** | % of packaged food and drinks classified as unhealthy according to the national food guide or dietary guidelines |
| **8** | % of packaged food and drinks classified as unhealthy according to the national nutrient profiling system |
| **9** | % of packaged food and drinks with a 'healthy score' according to the nutrient profiling system specific to the country |
| **10** | % of packaged food and drinks with an 'unhealthy score' according to the nutrient profiling system specific to the country |
| **11** | % of packaged food and drinks with the following degree of processing: unprocessed or minimally processed / processed culinary ingredients / processed / ultra-processed (NOVA) |
| **12** | % of packaged foods that meets government reformulation targets for risk nutrients (sodium, fat, sugar), by food category |
| **13** | % of packaged foods that meets WHO benchmarks for sodium |
| **14** | % of products (in a specific category) that exceed a global benchmark for nutrients to limit |
| **15** | Mean level of saturated fat (/100g or 100ml and per serve) |
| **16** | Mean level of total sugars (g/100g or 100ml and per serve) |
| **17** | Mean level of added sugars (g/100g or 100ml and per serve) |
| **18** | Mean level of sodium (mg/100g or 100ml and per serve) |
| **19** | Mean energy density (kJ or kcal/100g or 100ml and per serve) |
| **20** | Mean serving size (g or ml) |
| **21** | Mean level of trans fat (g/100g or 100ml and per serve) |
| **22** | Mean level of total fat (g/100g or 100ml and per serve) |
| **23** | Mean level of monounsaturated fat (g/100g or 100ml and per serve) |
| **24** | Mean level of polyunsaturated fat (g/100g or 100ml and per serve) |
| **25** | Mean level of carbohydrates (g/100g or 100ml and per serve) |
| **26** | Mean level of dietary fiber (g/100g or 100ml and per serve) |
| **27** | Mean level of proteins (g/100g or 100ml and per serve) |
| **FAST FOODS / RESTAURANT FOODS** | |
| **28** | % of foods available in major fast food chains with an 'unhealthy score' according to a nutrient profiling system |
| **29** | % of products (in a specific category) that exceed a global benchmark for nutrients to limit |
| **30** | % contribution of a product/combo meal to daily population Nutrient Reference Values (NRVs) for energy, sodium, sugar and saturated fat using as benchmarks  -8700kJ for energy (from Australian fast food supply report)  -2000mg for sodium (the WHO sodium upper limit)  -saturated + trans ≤10% energy per day based on 8700kJ diet   -sugar ≤10% energy per day based on 8700kJ diet |
| **31** | % of outlets that provide publicly available nutrition information for menu items |
| **32** | Mean level of saturated fat (/100g or 100ml and per serve) |
| **33** | Mean level of total sugars (g/100g or 100ml and per serve) |
| **34** | Mean level of sodium (mg/100g or 100ml and per serve) |
| **35** | Mean energy density (kJ or kcal/100g or 100ml and per serve) |
| **36** | Mean serving size (g or ml) |
| **37** | Mean level of trans fat (g/100g or 100ml and per serve) |
| **38** | Mean level of total fat (g/100g or 100ml and per serve) |
| **39** | Mean level of monounsaturated fat (g/100g or 100ml and per serve) |
| **40** | Mean level of polyunsaturated fat (g/100g or 100ml and per serve) |
| **41** | Mean level of added sugars (g/100g or 100ml and per serve) |
| **42** | Mean level of carbohydrates (g/100g or 100ml and per serve) |
| **43** | Mean level of dietary fiber (g/100g or 100ml and per serve) |
| **44** | Mean level of proteins (g/100g or 100ml and per serve) |

**FOOD LABELLING**

*(FOP = Front of pack)*

| **NUTRIENT DECLARATIONS** | |
| --- | --- |
|  | ***Packaged foods*** |
| **1** | Proportion of foods with a nutrient declaration (e.g., nutrition information panels) (by healthy vs less healthy foods) |
| **2** | Proportion of foods with a nutrient declaration in line with Codex standards (by healthy vs less healthy foods) |
| **3** | % products with nutrition information available in online retail settings |
|  | ***Restaurants*** |
| **4** | % of menu items in the fast food supply that have energy content or interpretative nutrition indicator |
| **5** | % of major fast-food chains that have adopted energy labelling across more than 80% of products |
| **LIST OF INGREDIENTS** | |
| **6** | % of foods with a list of ingredients (by healthy vs less healthy foods) |
| **7** | % of foods with quantitative ingredients information (QUID) (by healthy vs less healthy foods) |
| **SUPPLEMENTARY/VOLUNTARY NUTRITION INFORMATION** | |
| **8** | % of eligible products displaying supplementary nutritional information (e.g., traffic-light labelling of nutrient or foods, %GDA), by healthy vs less healthy foods |
| **9** | % of packaged food and drinks that display supplementary nutrition information, by healthy vs less healthy foods |
| **10** | Average level of healthiness of packaged foods with supplementary nutrition information present vs absent on label |
| **11** | % of foods with each different supplementary nutrition information scheme in use, by healthy vs less healthy foods |
| **12** | Median score [of the nutrient profiling system specific to the country] of packaged food and drinks carrying a voluntary FOP logo on the label |
| **13** | Median score [of the nutrient profiling system specific to the country] of packaged food and drinks NOT carrying a voluntary FOP logo on the label |
| **HEALTH AND NUTRITION CLAIMS** | |
| **14** | % of products featuring a claim (total claims, health claims, nutrition content claims) (by food categories and healthy vs less healthy foods) |
| **15** | % of foods making a claim that meets Codex standards (by healthy vs less healthy foods) |
| **16** | % of foods with nutrition claims referencing noncommunicable diseases (by healthy vs less healthy foods) |
| **17** | % of different types of nutrition claims on foods (by healthy vs less healthy foods) |
| **18** | % of different types of health claims on foods (by healthy vs less healthy foods) |
| **19** | % of packaged food products with nutrition claims on the FOP (by healthy vs less healthy foods) |
| **20** | % of packaged food products with health claims on the FOP (by healthy vs less healthy foods) |
| **21** | % of FOP nutrient content or health claims claims that did not fully comply with regulations |

**FOOD MARKETING**

| **ALL MEDIA AND SETTINGS** | |
| --- | --- |
| **1** | Total exposure to unhealthy food marketing per day |
| **2** | Estimated weekly exposure to unhealthy food marketing (weekly minutes) |
| **3** | Frequency of food ads with promotional techniques (e.g., child/teen/parental targeting, licensed characters, branded characters, etc.) |
| **TELEVISION** | |
| **4** | % of all ads on TV that were for unhealthy foods |
| **5** | % of food ads on TV that were for unhealthy foods |
| **6** | % of unique ads on TV that were for unhealthy foods |
| **7** | Mean rate/Frequency/% of unhealthy foods advertisements per channel per hour (during programming with >35% child audience) |
| **8** | Mean rate/Frequency/% of unhealthy foods advertisements per channel per hour (during children's peak viewing times and/or non-peak viewing times) |
| **9** | Mean rate of unhealthy food ads across the day (e.g., 6am to 10pm) on channels that have or are likely to have substantial child audiences |
| **10** | Mean rate of unhealthy food ads across the day (e.g., 6am to 10pm) on channels (regardless of audience) |
| **11** | Number/Duration of TV ads for unhealthy vs healthy food (in children’s peak viewing time and at all viewing times) |
| **12** | Proportion/Frequency (ads/h/channel) of all ads that were for food or beverage products |
| **13** | Average frequency of ads for foods that would be permitted to be advertised |
| **14** | Advertising gross rating points (GRPs)^1^ for children for unhealthy foods |
| **15** | Advertising impressions for ads with unhealthy foods (i.e., the number of times ads were viewed by all visitors/viewers combined) |
| **16** | Rate of product ads vs. brand ads for unhealthy foods/brands |
| **17** | Proportion of food advertisements by major food categories |
| **18** | Rate of food ads by food category (during peak and non-peak viewing times) |
| **19** | Rate of ads for food groups and indicator foods (over a broadcast period and/or peak viewing times/programmes) |
| **20** | Mean rate/Frequency of all advertisements per channel per hour (by peak viewing times children 5-12; non-peak viewing times; regulated children’s hours (am and pm); popular children’s programs) |
| **21** | Person-minute-views (PMVs) of all food advertising |
|  | ***Advertising impacts*** |
| **22** | Unhealthy food television advertising impacts (ads x views) for children 5-13 years during weekdays. |
| **23** | Unhealthy food television advertising impact (ads x views) for children 5-13 years during weekend days |
|  | ***Persuasive power*** |
| **24** | Number/%/Mean/Frequency/Rate of unhealthy ads featuring a promotional technique (all containing one or more, or by individual technique e.g., characters, premiums) |
| **25** | Mean rate of healthy vs unhealthy food advertisements per channel per hour with persuasive promotional techniques |
| **26** | % of health claims in ads that are for ‘unhealthy’ vs ‘healthy’ food and beverage products |
| **27** | % of nutrition claims in ads that are for ‘unhealthy’ vs ‘healthy’ food and beverage products |
| **28** | Top five food and beverage categories advertised to children (across a specific media or across all media) |
|  | ***Companies that advertised*** |
| **29** | Advertising parent companies contributing to 1% or more of overall food and beverage advertisements |
| **OUTDOOR** | |
| **30** | Number of unhealthy food ads in public transport settings like bus/train stations, bus/stops, bus shelters likely to be frequented by children (major routes to schools / city centres) |
| **31** | Density of total, unhealthy, healthy and miscellaneous food advertisements per 100m^2^ (within 500m or 250m from the school boundary)  -by urban and peri-urban areas  -by size of advertisements  -by use of promotional characters  -in high/medium and low SES areas  -in high- and low-density population areas |
| **32** | Median number of food and beverage ads within 250m around the school  -by school area: urban, peri-urban  -school fee level: low, medium, high  -school type: primary, secondary  -school category: government-funded, private |
| **33** | Mean rate of healthy vs unhealthy food advertisements around schools with promotional persuasive promotional techniques |
| **34** | Average number of outdoor advertisements for unhealthy vs healthy food and drinks en route to school, on public transport infrastructure, near schools |
| **35** | Number of unhealthy food ads in public transport settings like bus/train stations |
| **36** | % of food and drink outdoor advertising that promoted unhealthy products |
| **37** | Rate (and size) of overall ads within 250 m and 500 m from specific facilities (schools, recreation centres, etc.) |
| **38** | Rate (and size) of food vs. non-food ads within 250 m and 500 m from specific facilities (schools, recreation centres, etc.) |
| **39** | Rate (and size) of unhealthy food ads vs. healthy food ads within 250 m and 500 m from specific facilities (schools, recreation centres, etc.) |
| **40** | Rate (and size) of ads for food groups and indicator foods within 250 m and 500 m from specific facilities (schools, recreation centres, etc.) |
| **DIGITAL MEDIA** | |
|  | ***Social media*** |
| **41** | %/Number/Number per hour/Number per week of unhealthy food ads that children are exposed to (could use additional criteria of ads that are appealing to children and/or adolescents)   -on mobile devices  -on Facebook posts from popular packaged food, beverage and fast-food brands |
| **42** | Frequency of brand posts on social media (for largest food companies/predominantly unhealthy brands) |
| **43** | Marketing strategies used in social media brand posts |
| **44** | Number and % of Facebook posts from popular packaged food, beverage and fast-food brands with 100% of products classified as unhealthy |
| **45** | Number and % of Facebook posts from popular packaged food, beverage and fast-food brands that engaged in activities, promotional strategies and premium offers |
| **46** | Frequency and % of total mentions and total reach for the top 40 brands marketed to children, by food category on popular social media platforms over a year (user-generated posts; level of consumer interactions) |
| **47** | Average net sentiment scores of the top 40 brands marketed to children by food category on popular social media platforms over a year (user-generated posts; level of consumer interactions) |
| **48** | Total followers of Twitter users who have Tweeted about the top 40 brands marketed to children, and Twitter impressions of each mention by food category over a year (user-generated posts; level of consumer interactions) |
| **49** | Number and % of Likes, Shares and Comments per post and Views per video Facebook posts from popular packaged food, beverage and fast food brands (user-generated posts; level of consumer interactions) |
| **50** | Frequency (and %) of Twitter/Instagram/Facebook mentions and total reach (and %) for the top 40 brands marketed to children, by food category over a year (company posts) |
| **51** | Total Twitter followers and impressions the top 40 brands marketed to children, by food category (company posts) |
|  | ***Websites (e.g., popular websites for a target group, not including social media)*** |
| **52** | Frequency of food ads per site with promotional techniques used |
| **53** | Rate of product vs. brand ads per site |
| **54** | Frequency/Rate of unhealthy food ads vs. healthy food ads (per website) |
| **55** | Frequency of food vs. non-food ads |
| **56** | Frequency of ads for food groups and indicator foods |
| **57** | Frequency of advertisements for healthy vs. unhealthy foods by largest food companies |
| **58** | Rate of food ads by food group per site |
|  | ***Video games (console and online)*** |
| **59** | Rate per hour of paid for unhealthy food marketing on gaming platforms X screentime to estimate exposure per child. |
| **60** | Estimated exposure per child = Rate per hour of paid unhealthy food ads on gaming platforms X screentime |
| **61** | Number of brands mentions in chat room messages and message views (including sponsored ads + influencers) |
| **62** | Rate of product vs. brand ads (trimming amount of time of exposure measures per child) |
| **63** | Frequency of food ads with promotional techniques on gaming platforms (e.g., child/teen/parental targeting, licensed characters, branded characters, etc.) |
| **64** | Rate of unhealthy vs. healthy food ads on gaming platforms |
| **65** | Rate of food ads on gaming platforms by food group |
|  | ***Company websites*** |
| **66** | Number of marketing techniques used on food and beverage brand and company websites (targeting children/adolescents and general population) by type of marketing technique |
| **SPONSORSHIP & SPORTS SETTINGS** | |
| **67** | % of community sports clubs that have unhealthy food sponsors (in regional or metropolitan areas) |
| **68** | Frequency and rate (sponsors/organization)   -of overall sponsors  -of food vs. non-food sponsors  -of unhealthy food sponsors vs. healthy food sponsors |
| **69** | # of ads for unhealthy food at sports stadiums / community and professional recreation and leisure settings (brand or product based) |
| **70** | Sports-based food related marketing at and around sporting activity itself. |
| **71** | Rate of unhealthy vs. healthy food ads in sports settings |
| **72** | Rate of food ads by food group in sports settings |
| **73** | Rate of product vs. brand ads in sports settings |
| **74** | Frequency of food ads with promotional techniques in sports settings |
| **75** | % of food and beverage sponsors disclosed on sporting club websites |
| **76** | Types of sponsorship arrangements (e.g. logos on children’s uniforms, vouchers for products as rewards). |
| **PRODUCT PACKAGING** | |
| **77** | Frequency/Rate/% of food products (within different food groups)   -with promotions on packaging  -with promotions on packaging that are healthy vs healthy   -that are child-targeted that are healthy vs healthy |
| **78** | % of unhealthy products that have elements that appeal to children (within different food groups) |
| **79** | Types of promotions present on packaging |
| **SCHOOLS** | |
| **80** | % of schools with unhealthy food advertising |
| **81** | % of schools using (not using) food and beverage companies for sponsorship |
| **82** | Frequency and % of schools with a written, verbal or no food and beverage marketing policy |
| **83** | Frequency and % of schools with food or beverage advertisements on property |
| **84** | Frequency and % of schools with food or beverage displays on property |
| **85** | Frequency and % of schools with exclusive marketing arrangement |
| **86** | Frequency and % of schools participating in reward or incentive program |
| **87** | Frequency and % of schools distributing free branded items |
| **88** | Frequency and % of schools fundraising by selling branded items |
| **89** | Frequency and % of schools involved in sponsored competitions or contests |
| **90** | Frequency and % of schools receiving money in return for publicity |
| **91** | Frequency and % of schools having sponsored programs |
| **92** | Frequency and % of schools that had taken part in market research activities |
| **93** | Frequency and % of schools promoting/participating in scholarship programs |
| **94** | Frequency and % of schools with posted student made marketing materials |
| **PRINT** | |
| **95** | % of branded and non-branded references for healthy and unhealthy foods in magazines popular among children and adolescents 10-17 years |
| **96** | Frequency of unhealthy food ads vs. Healthy food ads in magazines popular among children |
| **97** | Frequency of ads for food groups and indicator foods in magazines popular among children |
| **98** | Types of promotion (e.g. direct ad, in editorial material, games) in magazines popular among children |
| **99** | Print space for food and unhealthy food ads (% of page, % of magazines) in magazines popular among children |
| **100** | Frequency of overall ads in magazines popular among children |
| **101** | Frequency of food vs. non-food ads in magazines popular among children |

1. “GRPs are used in advertising to measure the size of an audience reached by a specific media vehicle or schedule. Specifically in the TV advertising industry, ratings provide a percentage estimate of the size of the audience exposed to a TV advertisement. For example, if 500 audience members of 1000 viewed a certain TV advertisement, the advertisement would receive a rating of 50, meaning that it was seen by 50% of the TV audience.” ([Kim et al. 2012](https://academic.oup.com/heapro/article/28/1/17/578626?login=false))

**FOOD PRICES**

| **FOOD COSTS** | |
| --- | --- |
| **1** | Changes in the cost of healthier and less healthy foods found within the CPI over time |
| **2** | Changes in the cost of healthier and less healthy foods found within a basket of foods commonly consumed over time |
| **3** | Prices of different food groups (minimum number) and then all the foods healthy vs. less healthy over time |
| **4** | Changes in the price of minimally processed vs. ultra-processed foods over time |
| **5** | Changes in the cost of different food groups (not item specific but food group specific) over time |
| **6** | Prices of healthy vs unhealthy foods within food categories |
| **7** | Trend in price changes over time for healthy vs less healthy food and for minimally processed, processed, and ultra-processed food |
| **8** | Differential between the price of selected ‘healthy’ foods and ‘less healthy’ foods |
| **9** | Change in the price of basket of standard ‘less healthy’ foods over time |
| **10** | Average cost of fruits and vegetables vs. cost of starchy staples |
| **11** | Average cost of sodas vs. cost of starchy staples |
| **12** | Actual and relative price differential between each degree of processing food group (or ultra-processed vs. minimally processed foods) |
| **13** | Cost of vegetables in purchasing power parities (PPP) dollar/capita/day |
| **MEAL COST** | |
| **14** | Cost of popular take-away vs home-made meals |
| **15** | Differential between the price of ‘healthy’ and ‘less healthy’ meals |
| **DIET COST** | |
| **16** | Differential between the price of ‘healthy’ (recommended) and ‘less healthy’ (current) diets / Cost of a healthy diet relative to the cost of current diet   -for different ethnic groups  -by SES and area-level remoteness  For various sized households: 4 persons household; Single person households; Single parent household; Multiple households living together; Household compositions can differ) |
| **17** | Cost per person per day of a current diet and a healthy diet (in terms of least-cost food baskets) |
| **18** | Cost per person per day of a current diet and a healthy diet by food group (starchy staples, protein-rich foods, dairy, fruits, vegetables, fats |
| **19** | Cost per person per day of a nutrient adequate diet as the lowest-cost set of items available at each time and place that would stay within the low and upper bounds for dietary energy and all essential nutrients (Diet that meets harmonized dietary guidelines. Average household of 4 people.) |
| **20** | Cost per person per day of an energy sufficient diet (in terms of least-cost food baskets) |
| **21** | Cost per person per day and/or per household per fortnight of a healthy diet or diet meeting food-based dietary guidelines (in terms of least-cost food baskets) (by area-level SES and remoteness) |
| **22** | Cost of a healthy diet extension (capturing food preferences) or Nutritional Price Index (which includes all foods from the country's consumer price index (CPI) adjusted such as the weight of items in each food group meets dietary guidelines, while preserving quantities of items) |
| 23 | Cost of a healthy diet relative to the cost of sufficient energy from starchy staples |
| **24** | Regionally comparable estimates of diet costs ($US per day) for a standardised set of healthy and sustainable dietary patterns (flexitarian diet, pescatarian diet, vegetarian diet, vegan diet, diet variant high in fruits and vegetables, high-grain diet variant) based on current food prices. |
| **25** | Relative % changes in diet costs (flexitarian diet, pescatarian diet, vegetarian diet, vegan diet, diet variant high in fruits and vegetables, high-grain diet variant) when considering external cost estimates – cost of diet-related illness and diet-related costs of climate change |
| **AFFORDABILITY** | |
| **26** | Cost of diets (energy sufficient, nutrient adequate or healthy) compared with the international poverty cut-offs |
| **27** | Ratio of the cost of diets (energy sufficient, nutrient adequate or healthy) and average national food expenditures per capita |
| **28** | Cost of healthy diet and the cost of a basic diet as a proportion of income of reference household or relative to total income (by income level and area-level remoteness)   - Basic diet - ‘basic groceries’ - Affordable - accessible diet. - Healthy diet = exemplary diet - Healthy diet with the cheapest brands to assess affordability |
| **29** | Percentage of income required to purchase a healthy and a current diet |
| **30** | Number of people who cannot afford a healthy diet |
| **31** | % of the population who cannot afford a healthy diet |
| **32** | Cost of nutrient adequacy as a percent of household food expenditure |
| **33** | Cost of nutrient adequacy in local currency units |
| **FOOD PRICE PROMOTIONS** | |
| **34** | Average discount on unhealthy versus healthy foods (also under Retail) |
| **FOOD EXPENDITURE** | |
| **35** | Current spending on unhealthy food and drinks as a proportion of total amount spent on food (proxy measure of dietary intake than food prices) |
| **36** | Current spending on restaurant/ out-of-home foods |

**FOOD PROVISION**

| **GENERAL (PUBLICLY FUNDED SETTINGS)** | |
| --- | --- |
| **1** | % of public institutions that have a healthy food (or nutrition) policy |
| **2** | % of public institutions that have implemented a nutrition policy or programme |
| **3** | % of public institutions for which an *enforceable* food policy is in place |
| **4** | % of public institutions in which fresh vegetables are provided or are available for sale every day |
| **5** | % of public institutions complying with a nutrition policy or programme |
| **6** | % of foods provided and sold in public institutions meeting food group- or nutrient-based standards |
| **7** | % of public institutions complying with their healthy food (or nutrition) policy |
| **8** | % of foods within key food categories to encourage and/or limit that were compliant with food-based standards |
| **9** | Qualitative assessment of food products relative to food-based and/or nutrient-based standards |
| **SCHOOLS** | |
|  | ***Food availability*** |
| **1** | % of schools reporting selling food and beverages to students during the school day |
| **2** | % of schools with canteen/food service menus that had a minimum of 60% healthy food and drinks (as per corresponding government policy or relevant nutrient profiling system) |
| **3** | % of schools with canteens/food services that offered unhealthy food or drinks including , even on an occasional basis (corresponding policy states no unhealthy food should be available) |
| **4** | % of schools with vendors for competing foods (private parties, schools, vending machines, stores) outside of school feeding programs that offer unhealthy foods |
| **5** | % of schools offering only milk and water for sale to students during the school day (as recommended per the country Ministries of Health and of Education) |
| **6** | % of food groups offered for sale that were healthy, less healthy and unhealthy, by school area-level SES |
| **7** | % of schools that did not offer any ‘healthy’ and 'unhealthy' food groups for sale, by school area-level SES |
| **8** | % of schools that comply with government policies |
| **9** | % of schools that sell (or do not sell) sugar-sweetened beverages, by school area-level SES |
| **10** | % of healthier beverage options provided or sold out of total beverages available |
| **11** | Ratio of healthy vs. unhealthy beverage options sold or provided |
| **12** | % of schools in which sugary drinks are provided or are available for sale more than once a week |
| **13** | % of schools that do not sell or provide sugary drinks, excluding on special occasions) |
| **14** | % of schools that do not sell sweet, salty or deep-fried foods |
| **15** | % of schools in which fried food is provided or is available for sale more than once a week |
| **16** | % of schools in which salty snacks are provided or are available for sale more than once a week |
| **17** | % of schools in which salty snacks are provided or are available for sale more than once a week |
| **18** | % of schools in which fresh fruit is provided or is available for sale every day |
| **19** | % of schools with sufficient access to safe drinking water that is regularly assessed for quality |
| **20** | % of schools with access to *safe drinking water* at any time |
|  | ***Sources of foods*** |
| **23** | Of schools that provide a school meal or snack, the % of schools that provide healthy and nutritious foods and drinks |
| **24** | % of schools that source foods for school meals and snacks from local supply chains |
| **25** | Most common sources of selling food (3 most common, e.g., lunch order-in system, canteen run by school, etc.) |
| **26** | % of schools having ≥1 vending machines on school grounds |
|  | ***Healthy food policy*** |
| **27** | % of schools with canteens/food services that met all policy criteria |
| **28** | Average score (out of 100%) for strength and comprehensiveness of school nutrition policies |
| **29** | % of schools with a written nutrition policy |
|  | ***Fundraising activities*** |
| **30** | Number (%) of schools using food and beverages for fundraising |
| **31** | Number (%) of schools using ‘occasional’ foods in fundraising |
| **32** | Mean % of items used for fundraising in schools that are ‘healthy’ and 'unhealthy' food and beverage items |
|  | ***Food and nutrition programmes*** |
| **33** | % of schools participating in a food provision programme (school food programme) |
| **34** | % of schools participating in a nutrition programme (e.g., Enviroschools or Heart Schools in NZ) |
|  | ***Nutrition-related activities*** |
| **35** | % of schools that are free from unhealthy food marketing and sponsorship |
| **36** | % of schools that are free from marketing for unhealthy foods and related brands |
| **37** | % of school that have a garden that is being actively used (% of schools that have a kitchen garden for students to use ) |
| **38** | % of school that have a nutrition education in the curriculum |
| **39** | % of school reporting obstacles to improving their food and nutrition environment |
| **HOSPITAL AND HEALTHCARE SETTINGS** | |
|  | ***Food availability*** |
| **40** | % of food and drinks classified as ‘healthy’ (by outlet, vending machine) |
| **41** | % of food and drinks classified as ‘least healthy' (by outlet, vending machine) |
| **42** | % of food outlets that do not sell sugar-sweetened drinks |
|  | ***Healthy food policy*** |
| **43** | % of outlets that comply with all policy requirements (by outlet, vending machine) |
| **44** | % of outlets or vending machines compliant with specific policy requirements (by availability, product size, promotion and product quality practices) |
| **45** | % of red, amber and green foods (as per the national Healthy food and drink policy) by hospital and for vending machines and canteens/outlets separately |
| **46** | Average score for strength and comprehensiveness of national hospital nutrition policies |
| **SPORT AND RECREATION CENTRES** | |
|  | ***Food availability*** |
| **47** | % of sport and recreation centres that sold sugar sweetened beverages |
| **48** | % of items in snack vending machines that are ‘healthy’ and ‘less healthy’ snacks |
| **49** | % of items in beverage vending machines that are ‘healthy’ and ‘less healthy’ beverages |
| **50** | % of food products offered in concessions that are that are ‘healthy’ and ‘less healthy’ |
| **51** | % of beverages offered in concessions that are ‘healthy’ and ‘less healthy’ |
|  | ***Healthy food policies*** |
| **52** | % of sport and recreation centres reporting having a nutrition policy |
| **53** | % of sport and recreation centres reporting having a written nutrition policy |
|  | ***Promotions*** |
| **54** | Average number of promotions inside recreation centres for unhealthy foods |
| **55** | % of recreation centres that have exclusive vending contracts with major food companies |

**FOOD RETAIL**

| **FOOD RETAIL OUTLET MAPPING** | |
| --- | --- |
|  | ***Geographic food availability*** |
| **1** | Ratio of unhealthy food outlets to healthy food outlets (by region) |
| **2** | Change in ratio of unhealthy food outlets to healthy food outlets over time (by region) |
| **3** | Density of key food outlets per geographic area/population, overall and within specific neighbourhoods (e.g. rural/urban, socioeconomic) |
| **4** | Relative density (ratio of unhealthy to healthy) food outlets per geographic area/population |
| **5** | Density of key food outlets around residents’ homes and schools, overall and within specific neighbourhoods (e.g. rural/urban, socioeconomic) |
| **6** | Average density of unhealthy food outlets in neighbourhoods per 10,000 people in census areas (Most vs Least deprived areas)  - Food outlets classified as unhealthy: fast food, takeaway and convenience (bakery, confectionery store, dairy, service station) outlets. |
| **7** | Average density of unhealthy food outlets within 500m of schools, by urban/rural area and quintile of school socioeconomic deprivation  - Food outlets classified as unhealthy: fast food, takeaway and convenience (bakery, confectionery store, dairy, service station) outlets. |
| **8** | Average density of convenience stores and fast food + takeaway outlets per 10,000 people in census areas (all areas and within school zones), by SES |
| **9** | Average density of supermarkets and fruit and vegetable stores per 10,000 people in census areas, by SES |
| **10** | Average density of different types of food suppliers per 10,000 people in census areas, by SES |
| **11** | Relative density of healthy versus unhealthy food outlets around homes and schools |
|  | ***Overall food availability*** |
| **12** | Growth (% change) in number of modern grocery retailers^1^, over 1 (or 5 year), adjusted for population (e.g., change in number of stores per XX population) |
| **13** | Growth (% change) in number of supermarkets^1^, over 1 (or 5 year), adjusted for population (e.g., change in number of stores per XX population) |
| **14** | Growth (% change) in number of fast-food outlets, over 1 (or 5 year), adjusted for population (e.g., change in number of stores per XX population) |
| **15** | Growth (% change) in number of wet/fresh/farmers' markets, over 1 (or 5 year), adjusted for population (e.g., change in number of stores per XX population) |
| **16** | % change in the number of modern grocery stores over time – i.e., hypermarkets, supermarkets, discounters, forecourt retailers (grocery outlets attached to gas/petrol stations), and convenience stores |
| **17** | Number of modern grocery retailers per 100,000 population |
| **18** | Number of forecourt (petrol station) per 100,000 population |
| **19** | Number of convenience stores per 100,000 population |
| **20** | Number of supermarkets per 100,000 people |
| **21** | Number of fast-food outlets per 100,000 people |
| **22** | Number of wet/fresh/farmers' markets per 100,000 people |
| **SUPERMARKETS** | |
|  | ***Food labelling*** |
| **23** | Proportion of own-brand products that display a supplementary nutrition information (SNI) scheme (by supermarket chain) and change over time |
|  | ***Price promotion*** |
| **24** | % of unhealthy foods on price promotion vs proportion of healthy foods on price promotion each week |
| **25** | Average discount on unhealthy vs healthy foods (in weekly flyers) |
| **26** | % of price-promoted drinks that were for sugar-sweetened beverages |
| **27** | % of price-promoted shelf space in-store devoted to unhealthy food or drinks (by checkouts, end-of-aisle displays, island bins) |
|  | ***Product placement*** |
| **28** | % of checkouts where at least one type of unhealthy food or drink was present |
| **29** | % of end of aisle displays where at least one type of unhealthy food or drink was present |
| **30** | % of display space devoted to unhealthy foods and drinks at checkouts (for supermarkets and in convenience stores) |
| **31** | % of display space devoted to unhealthy foods and drinks in end-of-aisle displays (for supermarkets and in convenience stores) |
| **32** | % of junk-food free checkouts, by SES |
| **33** | % of junk food free endcaps, by SES |
|  | ***Shelf-space*** |
| **34** | % of shelf space allocated to unhealthy foods and drinks (compared with healthy items), between the most and least disadvantaged areas |
| **35** | Ratio of shelf-length of selected healthy: unhealthy food and beverages, by SES |
| **36** | Shelving length (or cm2) for each healthy product type in total and by visibility strata (i.e., low, medium or high visibility) |
| **37** | Shelving length (or cm2) for all healthy product types in total and by visibility strata |
| **38** | Shelving length (or cm2) for each unhealthy product type in total and by visibility strata |
| **39** | Shelving length (or cm2) for all unhealthy product types in total and by visibility strata |
| **40** | Ratio of all unhealthy to all healthy products by visibility strata |
| **41** | Aggregate availability of healthy and unhealthy foods (cumulative shelf space within predefined areas) |
|  | ***Catalogues*** |
| **42** | % of foods advertised in weekly catalogues that were unhealthy vs healthy (mail or online) |
|  | ***Advertising*** |
| **43** | % of junk-food free advertising (at entrance, in store windows, flyers), by SES |
| **44** | % of stores compliant with in-store advertising policies, guidelines or codes of voluntary practice |
|  | ***Food composition*** |
| **45** | Average score [of the nutrient profiling system specific to the country] of own-brand product portfolio (by supermarket chain) and change over time |
| **FAST FOOD CHAINS** | |
|  | ***Food*** ***composition*** |
| **46** | Average score [of the nutrient profiling system specific to the country] of manufacturer's product portfolio |
|  | ***Food availability*** |
| **47** | % of outlets for which sugar-sweetened beverages represent less than 50% of drink options on the menu of takeaway outlets |
| **48** | Number of street food vendors within 500m of main transport hubs [Street food] |
| **49** | % of street food items offered that is healthy [Street food] |
|  | ***Promotions*** |
| **50** | % of food/meals promoted inside outlets that were unhealthy |
|  | ***Compliance to policy*** |
| **51** | Evaluation of compliance with local policies, guidelines or codes of voluntary practice |
| **OTHER** | |
| **52** | % of products on home page of online grocery store websites or applications that are healthy [Online grocery store] |
| **53** | % of products/food outlets on home screen of online food delivery applications that are healthy [Food delivery apps] |

^1^ Definition provided on [The Food Systems Dashboard (2020)](https://www.foodsystemsdashboard.org/)

**FOOD TRADE AND INVESTMENT**

| TRADE IN GOODS | |
| --- | --- |
| 1 | Provisions in text relating to rules of origin |
| 2 | Import food volumes for key food categories |
| 3 | Rate of change in total food import volumes |
| 4 | Rate of change in key food category import volumes |
| 5 | Tariff-rate quotas for key food categories |
| 6 | Differential between tariffs on healthy and unhealthy key food categories |
| 7 | Food import volumes, by category |
| 8 | Rate of change in food import volumes, by category |
| 9 | Bound tariff rates for key food categories |
| 10 | Actual/applied tariff rates for key food categories |
| 11 | Tariff-rate quotas for all food categories |
| 12 | Tariff differential (if any) between all healthy and unhealthy key food categories |
| 13 | Retail food prices (in key food categories or more broadly where possible) |
| 14 | Retail food sales (in key food categories or more broadly where possible) |
| 15 | Population consumption volumes (in key food categories or more broadly where possible) |
| INVESTMENT & TRADE IN SERVICES | |
| 16 | Provisions in text relating to service sector liberalisation |
| 17 | Provisions in text relating to foreign capital constraints |
| 18 | Provisions in text relating to intellectual property rights (geographical indicators, trademarks) |
| 19 | Provisions in text relating to investor protections |
| 20 | Provisions in text relating to performance requirements |
| 21 | Type and country of origin of all foreign-owned transnational food corporations (TFCs) operating in country |
| 22 | FDI investment in food production, processing, retail and advertising sectors (monetary value) |
| 23 | Rate of change in total inward FDI in food and related sectors (including communications and advertising) |
| 24 | Market share of foreign TFCs in food production, processing, retail, and advertising sectors |
| 25 | Size of processed food sector |
| 26 | Rate of change in size of processed food sector |
| 27 | Degree of foreign concentration in food production, processing, retail, and advertising sectors |
| 28 | Environmental certifications of foreign-owned TFCs (e.g. Cradle to Cradle) |
| 29 | Domestic production (monetary value) of focus food categories or more broadly where possible |
| 30 | Changes in domestic policy relating to foreign ownership and investment |
| DOMESTIC PROTECTIONS AND SUPPORTS | |
| 31 | Provisions in text relating to safeguards and supports for agricultural products |
| 32 | Provisions in text relating to agricultural anti-dumping and countervailing measures |
| 33 | Provisions in text relating to agricultural export subsidies and promotion |
| 34 | Focus on a minimum of ten key exported food cateofires before the implementation of relevant agreements and assess their changes over time |
| 35 | Differential in subsidies between healthy and unhealthy key food categories |
| 36 | Export subsidies in key food categories |
| 37 | Total domestic production of selected key foods |
| 38 | Domestic production volumes of key food categories |
| 39 | Export volumes of key food categories |
| 40 | Rate of change of selected key foods between the proportions of food supply that is exported and that which is available for local consumption |
| POLICY SPACE AND GOVERNANCE | |
| 41 | Provisions in text relating to the protection of health and environment in the preamble |
| 42 | Provisions in text relating to general and specific exceptions for health and environment |
| 43 | Provisions in text relating to exclusions for health and environment |
| 44 | Provisions in text relating to basic rights for health and environment |
| 45 | Provisions in text relating to international standards for health and environment |
| 46 | Provisions in text relating to health and environment as legitimate policy objectives |
| 47 | Provisions in text relating to greater certainty around health and environmental policy |
| 48 | Provisions in text relating to objectives and principles related to health and environment |
| 49 | Provisions in text relating to good regulatory practices |
| 50 | Provisions in text relating to government procurement |
| 51 | Provisions in text relating to transparency |
| 52 | Provisions in text relating to state-state dispute settlement |
| 53 | Provisions in text relating to investor-state dispute settlement |
| 54 | Informal challenges to nutrition-related food policies (TBT committee) |
| 55 | Formal challenges to nutrition-related food policies (state-state dispute settlement) |
| 56 | Formal challenges to nutrition-related food policies (investor-state dispute settlement) |
| 57 | Changes in domestic policy, regulations and guidelines relating to food marketing composition and labelling |

**COMPANY LEVEL MEASURES** (across various settings)

| **COMPANY-LEVEL** | |
| --- | --- |
|  | ***Marketing*** |
| **1** | # of infractions per company for violating marketing regulations or voluntary industry-led codes |
| **2** | Food company marketing expenditures in various media/settings |
| **3** | Food company marketing expenditures by brand |
| **4** | Food company marketing expenditures by healthfulness classification |
| **5** | Frequency of food company marketing activities in various media/settings |
| **6** | Broadcaster revenue |
|  | ***Composition*** |
| **7** | Food company product range changes (purchase or sale of major brands, classified as healthy or less healthy) |
| **8** | Food sales by brand within a company portfolio of major food companies |
| **9** | Food sales by healthfulness classification, by company |
| **10** | % of products reformulated by food companies (to meet standards or targets) |
| **11** | % of products that comply with company-developed nutrition standards |
