# Supplementary material for: Prioritising Indicators for Large-scale Monitoring and Assessment of Food Environments for Public Health
Source: Curr Obes Rep. 2026 Apr 22;15(1):37. doi: 10.1007/s13679-026-00705-8 (PMC13102733; doi:10.1007/s13679-026-00705-8)
Supplement: Supplementary file 2 — Supplementary Material 2 (DOCX 26.0 KB) [file 13679_2026_705_MOESM2_ESM.docx]

**Online Resource 2 –** Result from online prioritisation exercise with food environments experts on priority indicators for large-scale monitoring and assessment in Step 3, showing the number of participants that assigned any rank, and the distribution of rankings for each indicator (from 1 to 7) * (n=51).

**Rank assigned**

*Some indicators that were part of the initial ranking exercise are not shown because they were subsequently removed from the short‑list during the study’s iterative review. In the figure, the number of participants who assigned a rank to each indicator varies, depending on how many participants included the indicator in their top seven indicators. Darker colours correspond to higher ranks and lighter colours correspond to lower ranks.

** Refer to Table 1 for a description of each indicator.
